# Oncolytic viruses alter the biogenesis of tumor extracellular vesicles and influence their immunogenicity

**DOI:** 10.1016/j.omton.2024.200887

**Published:** 2024-09-26

**Authors:** Ugo Hirigoyen, Coraly Guilbaud, Morgane Krejbich, Morgane Fouet, Judith Fresquet, Bastien Arnaud, Emmanuelle Com, Charles Pineau, Gwenann Cadiou, Julien Burlaud-Gaillard, Philippe Erbs, Delphine Fradin, Nathalie Labarrière, Jean-François Fonteneau, Tacien Petithomme, Nicolas Boisgerault

**Affiliations:** 1 Nantes Université, Inserm UMR 1307, CNRS UMR 6075, Université d'Angers, CRCI2NA, 44000 Nantes, France; 2 LabEx IGO, Nantes Université, 44000 Nantes, France; 3 University Rennes, Inserm, EHESP, Irset (Institut de recherche en santé, environnement et travail) – UMR_S 1085, 35000 Rennes, France; 4 University Rennes, CNRS, Inserm, Biosit UAR 3480 US_S 018, Protim core facility, 35000 Rennes, France; 5 Nantes Université, Inserm UMR 1302, CNRS EMR 6001, Université d’Angers, INCIT, 44000 Nantes, France; 6 Plate-Forme IBiSA de Microscopie Electronique, Université de Tours and CHRU de Tours, 37000 Tours, France; 7 Transgene SA, 67400 Illkirch-Graffenstaden, France; 8 Nantes Université, CHU Nantes, 44000 Nantes, France

**Keywords:** MT: Regular Issue, oncolytic viruses, extracellular vesicles, vesicular stomatitis virus, vaccinia virus, t cell, intercellular communication, immunogenicity, immunotherapy

## Abstract

Extracellular vesicles (EVs) are mediators of intercellular communication in the tumor microenvironment. Tumor EVs are commonly associated with metastasis, immunosuppression or drug resistance. Viral infections usually increase EV secretion, but little is known about the effect of oncolytic viruses (OVs) on tumor EVs. Here, we investigated the impact of oncolytic vesicular stomatitis virus (VSV) and vaccinia virus on EVs secreted by human melanoma and thoracic cancer cells. We found that OV infection increases the production of EVs by tumor cells. These EVs contain proteins of viral origin, such as VSV-G, thus creating a continuum of particles sharing markers of both canonical EVs and viruses. As such, the presence of VSV-G on EVs improves the transfer of their protein content to cell types commonly found in the tumor microenvironment. A proteomic analysis also revealed that EVs-OV secreted during VSV infection are enriched in immunity-related proteins. Finally, CD8^+^ T cells incubated with EVs-OV from infected cells display slightly enhanced cytotoxic functions. Taken together, these data suggest that OVs enhance the communication mediated by tumor EVs, which could participate in the therapeutic efficacy of OVs. These results also provide rationale for engineering OVs to exploit EVs and disseminate therapeutic proteins within the tumor microenvironment.

## Graphical abstract

**Figure:**
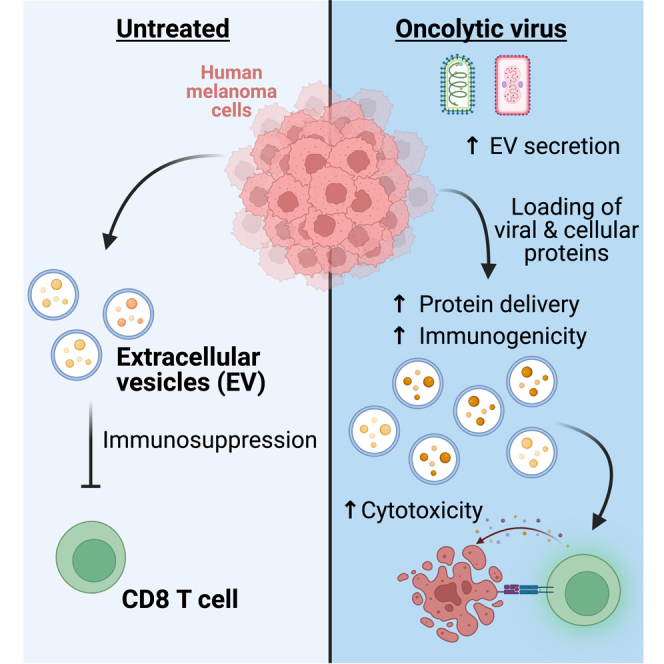

## Teaser

Boisgerault and colleagues investigated how OVs, which infect tumor cells and induce systemic antitumor immunity, modify the biology of tumor EVs. Their findings indicate that oncolytic infection increases the immunogenicity of EVs, thus suggesting that those could play a role in the therapeutic efficacy of oncolytic virotherapy.

## Introduction

Extracellular vesicles (EVs) are submicrometric particles secreted by all cell types that mediate intercellular communication. EVs are usually separated into different subtypes depending on their size: apoptotic bodies, large EVs and small EVs (sEVs).^1^ sEVs measure between 30 and 150 nm and can be further classified depending on their biogenesis pathway: exosomes correspond with EVs that are formed by inward budding of early endosomes, whereas ectosomes directly bud from the plasma membrane. sEVs are enriched in different protein markers, such as tetraspanins (CD9, CD63, and CD81), Programmed cell death 6-interacting protein (PDCD6IP, ALIX), or Tumor susceptibility gene 101 (TSG101), although there is no universal marker identified so far. Even though it remains unclear whether tumor cells secrete more EVs than their healthy counterparts, plasmatic EV levels are correlated with tumor burden.^2^^,^^3^ Tumor-derived EVs are often linked to mechanisms promoting oncogenesis and tumor progression.^4^^,^^5^^,^^6^ It has been shown that proteins and non-coding RNAs transported by EVs could impart oncogenic properties to other cells.^7^ These EVs also act on fibroblasts and endothelial cells and promote their pro-tumor properties, including drug resistance and metastasis.^7^^,^^8^^,^^9^ Factors delivered by tumor-derived EVs also modify the phenotypes and functions of immune cells, usually skewing them toward immunosuppression and promoting immune escape.^10^^,^^11^^,^^12^ In contrast, they can also exhibit immunostimulatory properties by transferring tumor-associated antigens to^13^^,^^14^ or by activating cGAS/STING signaling in^15^^,^^16^ dendritic cells (DCs).

Oncolytic viruses (OVs) specifically replicate in tumors and lyse malignant cells, which commonly induces an anti-tumor immune response.^17^^,^^18^ Two OVs—both herpes simplex virus 1 (HSV-1) strains—are currently approved for clinical use. Talimogene laherparepvec is approved by the U.S. Food and Drug Administration and the European Medicines Agency for the treatment of unresectable metastatic melanoma,^19^ and G47Δ recently obtained conditional approval in Japan for patients with glioblastoma after a successful phase II clinical trial.^20^ Other viruses are currently being evaluated in preclinical and clinical studies, such as the vesicular stomatitis virus (VSV), a member of the RNA virus *Rhabdoviridae* family, and the vaccinia virus (VACV), a member of the DNA virus *Poxviridae* family. There are several indications in the literature suggesting that EVs produced in the context of viral infections—including oncolytic infections—may impact the overall OV activity.^21^ First, viruses generally increase EV secretion because of the cellular stress they induce,^21^^,^^22^ and OVs and EVs have been shown to interact. Some studies report that oncolytic adenoviruses can be spontaneously packaged in tumor EVs,^23^^,^^24^ which could help virus propagation and promote a systemic therapeutic effect. Others were able to mimic this phenomenon and achieved systemic delivery of EV-encapsulated OVs.^25^^,^^26^^,^^27^ Finally, it was demonstrated that viral products conveyed by EVs can achieve therapeutic activity. Labani-Motlagh et al.^28^ observed that Tumor necrosis factor (TNF) superfamily proteins expressed by an engineered oncolytic adenovirus were present at the surface of EVs secreted by infected cells and could induce the maturation of DCs. Wedge et al. recently reported that microRNA (miRNA) or short hairpin RNA expressed by a recombinant VSV were transferred to uninfected tumor cells by EVs and could sensitize these cells to small molecule therapy and T cell killing.^29^ These studies and others^21^^,^^30^ highlight that viruses and EVs are not completely distinct entities. Indeed, infected cells secrete vesicles, which are similar to *bona fide* EVs, but are also loaded with proteins or nucleic acids of viral origin. Since these hybrid particles cannot be separated from *bona fide* EVs, in the present manuscript we use the terms “EVs-VSV” and “EVs-VACV” to refer to the vesicular secretome of cells infected by VSV or VACV, respectively.

Even if previous work has shown that EVs can contribute to the dissemination of either OVs or their payloads, how EV-mediated intercellular communication is affected by oncolytic infection remains to be elucidated. Here, we sought to understand how infection of human melanoma and thoracic cancer cells by oncolytic VSV or VACV would modify the phenotype of tumor EVs and alter the functions of cells exposed to those. We found that the oncolytic infection of tumor cells tends to enhance the intercellular communication mediated by EVs, that VSV induces an enrichment of immunity-related proteins in EVs and that these can increase the cytotoxicity of anti-tumor human CD8^+^ T cells.

## Results

### Tumor cells secrete more EVs when infected by OVs

**Figure 1:**
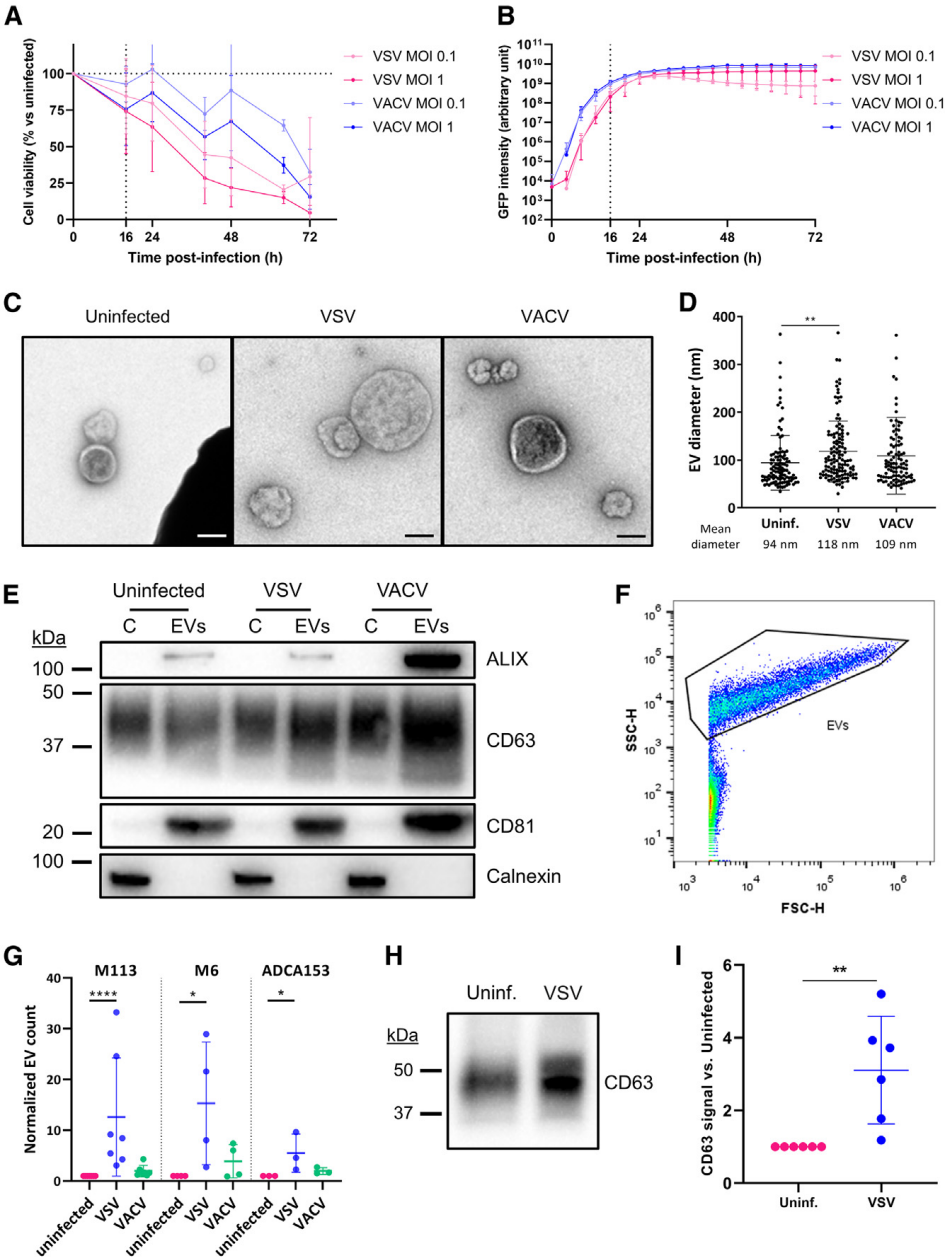
Tumor cells secrete more EVs upon OV infection (A and B) M113 melanoma cells were infected by VSV-GFP or VACV-GFP at a MOI of 0.1 or 1. Cell viability (A) and expression of the viral transgene (B) were measured over time. Dotted lines indicate when EVs were harvested in subsequent experiments. Data are presented as mean (SD). *n* = 3–4 biological replicates. (C) Representative transmission electron micrographs of EVs secreted by uninfected, VSV-infected or VACV-infected M113 melanoma cells. Scale bars, 100 nm. (D) Diameter of EV-shaped particles identified by TEM. Data are represented as mean (SD). *n* = 103–124 single EVs per condition. ∗∗*p* = 0.0032 (Kruskal-Wallis test). (E) Western blot analysis of EV (ALIX, CD81, and CD63) or cellular (calnexin) marker expression in cell lysates (C) or corresponding EV lysates (EVs) from uninfected, VSV-infected or VACV-infected M113 cells. Three μg of proteins were loaded in each lane. Representative of five independent experiments. (F and G) Single-EV flow cytometry analysis of EVs stained for tetraspanins (CD9/CD81/CD63). (F) EVs were gated based on the forward and side scatter parameters. (G) Relative quantification of CD9/CD63/CD81^+^ EVs secreted by M113, M6 melanoma, or ADCA153 lung adenocarcinoma cells. Data are presented as mean (SD). *n* ≥ 3 biological replicates. ∗∗∗∗*p* < 0.0001, ∗*p* = 0.0199 and 0.0189 for M6 and ADCA153, respectively (Kruskal-Wallis test). (H and I) Western blot analysis of EVs secreted by tumor cells, with equal volumes (10 μL) of EV lysates from uninfected or VSV-infected cells loaded in both lanes. Representative of 6 biological replicates. (I) Quantification of the CD63 signal on 6 biological replicates. Data are presented as mean (SD). ∗∗*p* = 0.0022 (Mann-Whitney test).

To investigate how infection by OVs alters EV biogenesis, we used either VSV (Indiana strain) or VACV (Copenhagen strain) to infect human melanoma cell lines. EVs were purified from culture supernatants 16 h after infection, before death of infected cells (Figures 1A, S1A, and S1B) and during the exponential phase of the viral transgene expression (Figures 1B and S1C). We first characterized the samples according to the recommendations of the International Society for Extracellular Vesicles.^31^ We validated the morphology of purified EVs by transmission electron microscopy (TEM) (Figure 1C) and found that the diameter of EVs-VSV is increased by about 25% compared with EVs derived from uninfected cells (Figure 1D). Supernatants were filtered before ultracentrifugation to remove VACV particles,^32^ but VSV was not completely eliminated. Indeed, TEM analyses showed that bullet-shaped objects (corresponding to VSV virions) represented approximately 28% of all observed objects (Figure S2A). Despite attempts to separate virions and EVs by flotation assay (Figures S2B–S2D) or size-exclusion chromatography (Figures S2E and S2F), we could not eliminate all infectious particles from EV preparations and this was taken into consideration for subsequent experiments. We observed that classical EV markers (ALIX, CD63, and CD81) are present in the preparations but not cellular calnexin (Figure 1E). We then used single EV flow cytometry (Figure 1F) to quantify EVs-OV secreted during infection. EVs-OV were stained for tetraspanins (CD9, CD63, and CD81) and we observed that cells infected by VSV—and to a lesser extent by VACV—produced more EVs-OV displaying at least one tetraspanin (Figure 1G). When analyzing equal volumes of EV preparations by western blot (Figures 1H and 1I), we showed that the EV markers CD63 is enriched during OV infection, thus confirming the results obtained by flow cytometry.

### Proteins encoded by OVs are packaged into EVs

**Figure 2:**
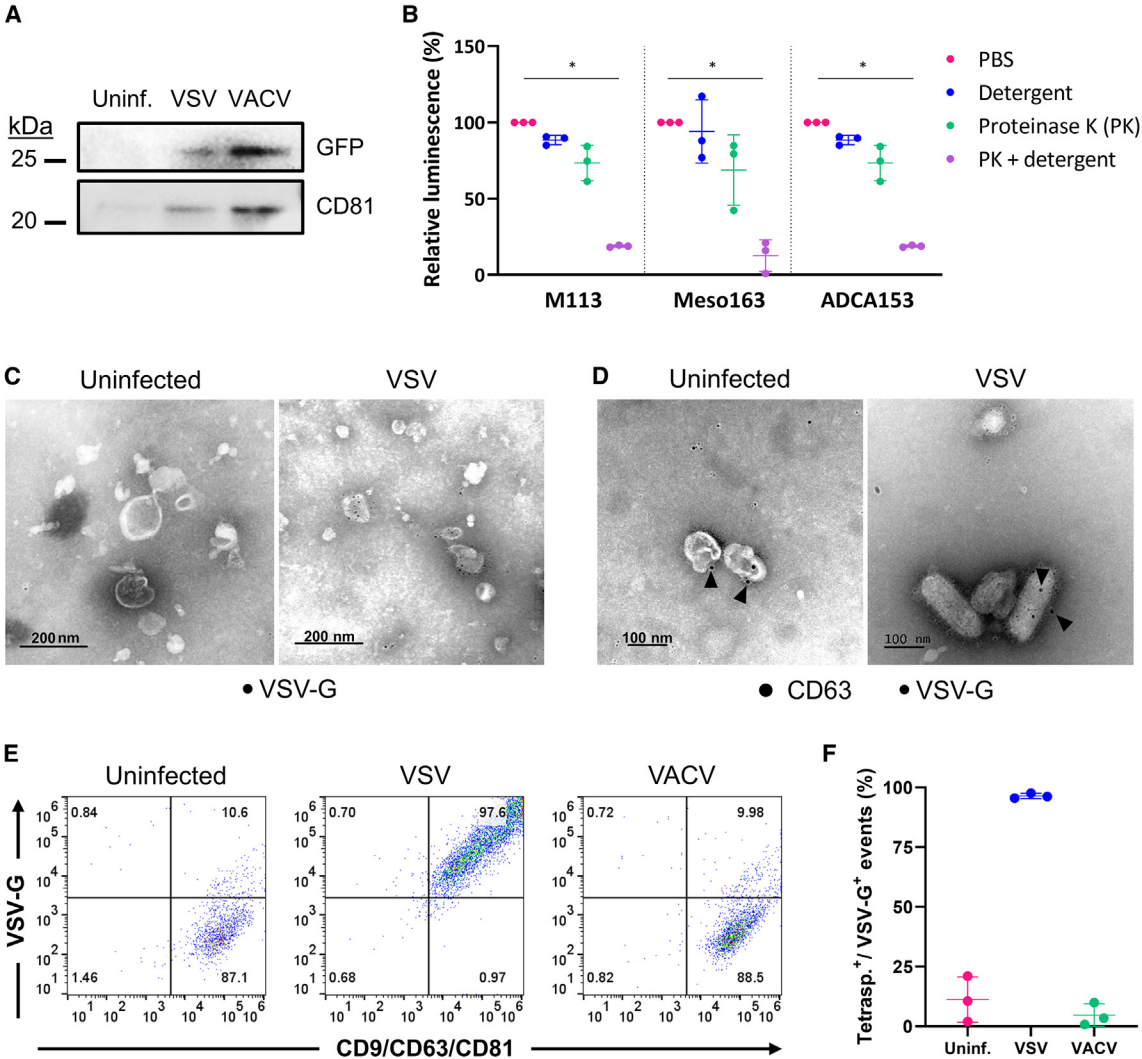
Tumor EVs spontaneously package proteins of viral origin (A) Western blot analysis of GFP and CD81 in EVs secreted by M113 cells infected by GFP-encoding VSV or VACV. Representative of three biological replicates. (B) Proteinase protection assay of EVs secreted by M113, Meso163 or ADCA153 cells infected by VSV-NLuc. EVs were incubated with either detergent, proteinase K or both before adding the NLuc substrate. Data are presented as mean (SD). *n* = 3 biological replicates. ∗*p* = 0.0125, 0.0372, and 0.0125 for M113, Meso163, and ADCA153, respectively (Kruskal-Wallis test). (C and D) Representative transmission electron micrographs of EVs secreted by uninfected or VSV-infected M113 cells and labeled with (C) an anti-VSV-G antibody coupled to 6-nm gold particles or (D) anti-CD63 (10-nm gold particles) and anti-VSV-G (6-nm gold particles) antibodies. Arrows indicate CD63 staining. (E) Single EV flow cytometry analysis (CD9/CD63/CD81 and VSV-G) of EVs secreted by uninfected, VSV-infected, or VACV-infected M113 cells. (F) Relative quantification of CD9/CD63/CD81/VSV-G^+^ EVs secreted by M113 cells. Data are presented as mean (SD). *n* = 3 biological replicates.

Consistent with previous findings,^28^ we found that the expression of OV-encoded transgenes is strong enough to allow spontaneous packaging of the recombinant proteins—here GFP—with EVs (Figures 2A and S3A). To determine whether these recombinant proteins are located inside EVs or simply associated with the outer layer of the membrane, we performed experiments using a detergent to dissociate lipid membranes. We showed that GFP was only accessible after detergent treatment of EVs-VSV (Figure S3B), thus demonstrating that GFP was originally inside EVs-VSV. We extended these results by using a VSV coding for another protein, the NanoLuc (NLuc) luciferase. In a proteinase-protection assay, EVs-VSV secreted by a panel of human and murine tumor cells infected by this virus were treated with either detergent, proteinase K or both (Figures 2B and S2C). As expected, the combination of proteinase K and detergent was necessary to quench the luminescence from the EVs, meaning that the NLuc was originally located inside EVs-VSV. This demonstrates that proteins encoded by OVs are spontaneously loaded into EVs.

Since viral proteins could have an impact on the tropism of EVs produced by infected cells, we then wondered whether those were also present in EVs. Focusing on the glycoprotein of VSV (VSV-G), we observed by TEM that EVs produced by VSV-infected cells were coated with VSV-G (Figure 2C). Surprisingly, we also found that VSV virions were not only VSV-G^+^ but also exhibited CD63 staining (Figure 2D). To quantify this phenomenon, we performed single EV flow cytometry experiments in which EVs-VSV were stained for both tetraspanins (CD9/CD63/CD81) and VSV-G (Figures 2E, 2F, and S4). We observed that close to 100% of EVs-VSV were double-positive, whereas EV preparations from uninfected cells or VACV-infected cells only stained positive for the tetraspanins. Overall, these results suggest that EVs-VSV present both viral and cellular markers on their surface, thus indicating that EVs-VSV may consist of a continuum of particles that share features of both virions and *bona fide* EVs.

### Viral material enhances EV-mediated intercellular protein transfer

**Figure 3:**
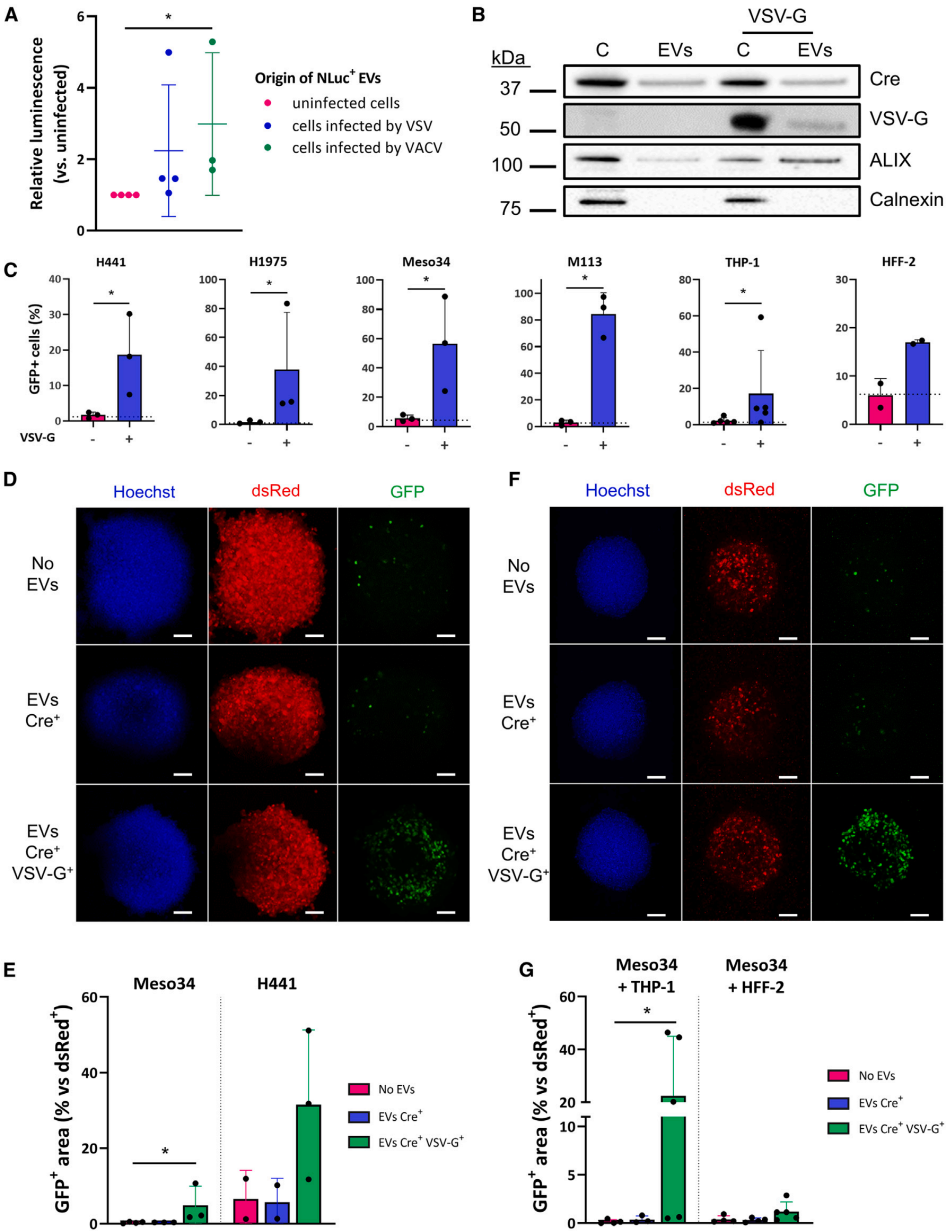
Viral material enhances EV-mediated intercellular protein transfer (A) EVs secreted by uninfected, VSV-infected or VACV-infected M113-NLuc cells were incubated for 4 h with parental M113 cells. EV internalization by recipient cells was measured by analyzing luminescence of target cells, normalized with input EV luminescence. Data are presented as mean (SD). *n* = 3 or 4 biological replicates. ∗*p* = 0.0115 (Kruskal-Wallis test). (B and C) EVs of Lenti-X 293T cells transfected to express Cre ± VSV-G were incubated with tumor cells transduced to express GFP upon Cre recombination. (B) Western blot analysis of Cre, VSV-G, ALIX and calnexin in purified EVs from transfected HEK cells. Three micrograms of proteins were loaded in each lane. Representative of two independent experiments. (C) Flow cytometry analysis of recipient cells incubated with Cre^+^ or Cre^+^/VSV-G^+^ EVs. The dotted lines indicate the background percentage of recipient cells expressing GFP. Data are presented as mean (SD). *n* = 2–4 biological replicates. ∗*p* = 0.05 for H441, H1975, Meso34, M113, HFF-2, and 0.0476 for THP-1 (Mann-Whitney test). (D) Confocal micrographs of tumor spheroids (loxP-dsRed/GFP-loxP H441 cells) incubated with mock, Cre^+^, or Cre^+^/VSV-G^+^ EVs. Images represent the maximum intensity *Z*-projections of all imaged slices. Scale bar, 100 μm. (E) Quantification of the GFP^+^ area in tumor spheroids (loxP-dsRed/GFP-loxP Meso34 or H441 cells) from (D). Data are represented as the ratio of GFP^+^ area over dsRed^+^ area within the same spheroid. *n* = 2–4 spheroids imaged per condition. ∗*p* = 0.0495 (Kruskal-Wallis test). (F) Confocal micrographs of MCTS (unlabeled Meso34 cells + loxP-dsRed/GFP-loxP THP-1 cells) incubated with mock, Cre^+^ or Cre^+^/VSV-G^+^ EVs. Images represent the maximum intensity *Z*-projections of all imaged slices. Scale bar, 100 μm. (G) Quantification of the GFP^+^ area in tumor spheroids (unlabeled Meso34 + loxP-dsRed/GFP-loxP THP-1 or HFF-2 cells) from (F). Data are represented as the ratio of GFP^+^ area over dsRed^+^ area within the same spheroid. *n* = 3–5 spheroids imaged per condition. ∗*p* = 0.0426 (Kruskal-Wallis test).

In physiological conditions, the transfer of functional intercellular EV cargo is a rare event.^33^ To investigate whether OV infection modifies the internalization of EVs, we first infected melanoma cells expressing NLuc, purified EVs 16 h after infection and added them onto recipient cells. After extensive washing to remove unbound EVs, we measured the luminescence, corrected by the input NLuc signal, to evaluate EV association with the target cells (Figure S5A). We observed an increase in luminescence when cells were incubated with EVs-VSV or EVs-VACV (Figure 3A), which suggests that OVs enhance the internalization of tumor EVs and the transfer of EV-carried cargos. This may be the consequence of the presence of viral proteins on the surface of EVs, at least for EVs-VSV, which facilitates their attachment and fusion, as it is well characterized for the glycoprotein VSV-G.^34^^,^^35^ To ensure that EVs have been internalized and have delivered their cargo to the cytosolic compartment of recipient cells, we used a Cre-mediated recombination assay,^8^ where EVs containing Cre are incubated with cells able to switch from dsRed to GFP expression upon Cre delivery and recombination (Figure S5B). To assess how the presence of viral proteins could modify the cargo delivery of EVs without using replication-competent VSV, we pseudotyped Cre^+^ EVs with VSV-G (Figure 3B). As expected, Cre^+^/VSV-G^+^ EVs led to a substantial recombination among recipient lung adenocarcinoma (H441 and H1975), mesothelioma (Meso34), melanoma (M113) cells, monocytes (THP-1) or fibroblasts (HFF2), whereas no GFP was detectable when Cre^+^ EVs without VSV-G were used (Figure 3C).

To study EV penetration in a three-dimensional (3D) environment, we then formed multicellular tumor spheroids (MCTS) with reporter mesothelioma and lung adenocarcinoma cells. We observed that Cre^+^/VSV-G^+^ EVs enabled recombination and GFP expression in these MCTS (Figure 3D) and could deliver their content to areas located beyond the outer cell layers of the spheroids (Figures S5C–S5E). However, this was not observed for all tested cells lines (Figure 3E) and was not correlated with what was observed in two-dimensional culture, which suggests that physical parameters may influence EV-mediated transfer in this context. To mimic a simplified tumor microenvironment, we also used complex MCTS containing both tumor cells and surrogates of healthy cells. In MCTS containing both unlabeled Meso34 mesothelioma cells and dsRed/GFP reporter THP-1 monocytic cells, we observed up to 40% of recombination among the latter (Figure 3F). VSV-G^+^ EVs seem to transfer their content more efficiently to THP-1 monocytes than HFF2 fibroblasts (Figure 3G). Altogether, our results suggest that VSV-G^+^ EVs from infected cells may be able to transfer their content more readily to uninfected cells, even in complex 3D environments, due to the presence of viral proteins on their surface.

### Infection by VSV leads to the loading of immunity-related proteins in EVs

**Figure 4:**
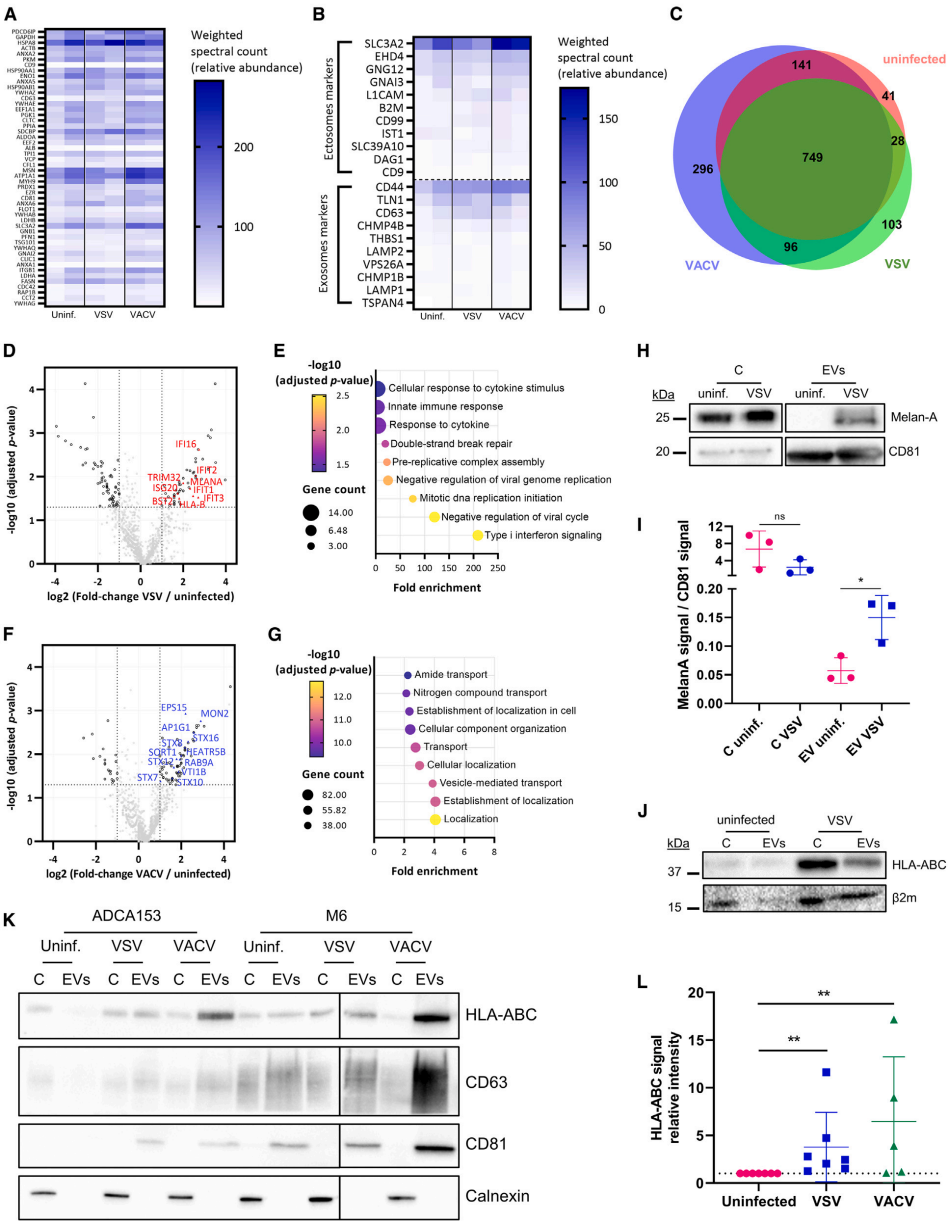
Exploratory MS screen identifies immunity-related proteins in EVs secreted by VSV-infected tumor cells (A–G) Liquid chromatography MS-based proteome analysis of EVs secreted by M113 cells. *n* = 2 biological replicates. (A and B) Relative quantification of proteins most commonly identified in Vesiclepedia^36^ (A) or annotated as ectosomes or exosomes markers^37^ (B). (C) Venn diagram of the identified proteins. (D) Volcano plot of proteins significantly enriched or depleted in EVs-VSV. Proteins encoded by IFN-stimulated genes are highlighted in red. (E) Significantly enriched Gene Ontology (GO) biological pathways in EVs-VSV. (F) Volcano plot of proteins significantly enriched or depleted in EVs-VACV. Proteins involved in endosomal transport are highlighted in blue. (G) Significantly enriched GO biological pathways in EVs-VACV. (H and I) Western blot validation of the enrichment of the melanoma antigen Melan-A in EVs-VSV secreted by M113 cells. (H) Representative experiment of 3 biological replicates. (I) Relative quantification of the Melan-A signal/CD81 signal in cell lysates (C) or EVs. *n* = 3 biological replicates. ∗*p* = 0.05. (J–L) Western blot validation of the enrichment of MHC class I molecules in EVs-VSV or EVs-VACV. (J) Detection of HLA-ABC and β2-microglobulin in EVs from uninfected and VSV-infected M113 cells. Three μg of proteins were loaded in each lane. Representative of 3 biological replicates. (K) Detection of HLA-ABC, CD63, CD81 and calnexin in EVs from uninfected and VSV-infected M6 or ADCA153 cells. Three micrograms of proteins were loaded in each lane. Representative of three biological replicates. (L) Relative quantification of the HLA-ABC signal in EVs-VSV and EVs-VACV from different cell lines compared with EVs from uninfected cells. *n* = 5–7 biological replicates. ∗∗*p* = 0.0039 and 0.084 for EVs-VSV and EVs-VACV, respectively (Kruskal-Wallis test).

To understand how OV infections modify the overall protein content of EVs and potentially their release in recipient cells, we then performed a proteomics analysis of EVs from human melanoma cells (Table S1). To validate the purity of the samples, we first confirmed that most of the top 50 proteins commonly identified in the *Vesiclepedia* database^36^ were enriched with satisfactory relative abundance scores (Figures 4A and S6A). We observed that markers commonly enriched in sEVs budding directly from the plasma membrane (ectosomes)^37^ were more abundant than markers associated with *bona fide* exosomes (Figure 4B). It is noteworthy that the infection had little effect on the balance between ectosomal and exosomal markers and that the EVs from the three conditions tested shared a large majority of the identified proteins (Figure 4C). As expected, we detected all five proteins encoded by the VSV genome, but also identified 29 proteins encoded by the VACV genome (Table S1), indicating either that some virions were co-purified with EVs despite filtration or that some viral proteins are packaged into EVs as shown in Figure 2. We found that EVs-VSV are enriched with proteins encoded by interferon (IFN)-stimulated genes (Figures 4D and 4E), which is consistent with an upregulation of the pathways related to the innate antiviral immune response in infected tumor cells. In contrast, EVs-VACV were not significantly enriched with immunity-related protein. Instead, enriched proteins were linked to intracellular trafficking and vesicular transport pathways (Figures 4F and 4G). For both viruses, the identified downregulated pathways were mostly related to cell cycle and mitosis (Figures S6B and S6C).

In addition to innate immunity proteins, we also identified proteins linked to the adaptive immune response in EVs-VSV (Figure 4D). Indeed, interesting hits from the proteomic analysis included the class-I presentation molecules human leukocyte antigen (HLA)-ABC and β2-microglobulin, but also the melanoma antigen Melan-A. The loading of Melan-A in EVs-VSV was validated by western blot (Figure 4H) and we confirmed that EVs-VSV contained higher quantities of Melan-A compared with EVs from uninfected melanoma cells (Figure 4I). Similarly, we confirmed that molecules from the major histocompatibility complex (MHC) class I complex were enriched in EVs-VSV and EVs-VACV purified from different tumor cell lines (Figures 4J–4L). Overall, this proteomics analysis shows that EVs-VSV are enriched in immunity-related proteins, in comparison with EVs secreted by uninfected cells.

### Tumor EVs secreted upon VSV infection partly enhance CD8^+^ T cell functions

**Figure 5:**
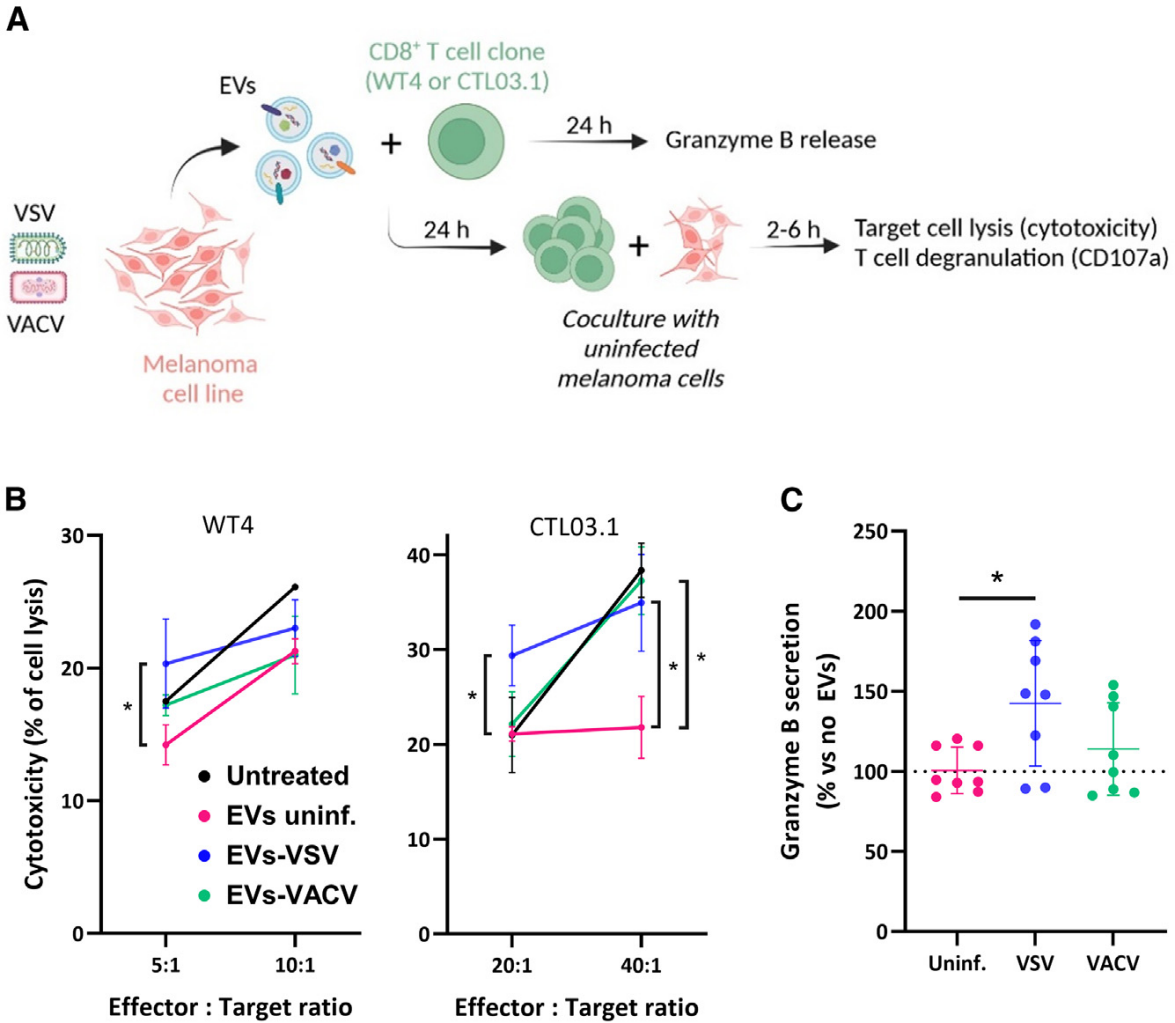
EVs secreted by OV-infected tumor cells partly enhance the cytotoxicity of anti-tumor CD8^+^ T cell clones (A) EVs secreted by M113 cells were incubated with the WT4 or CTL03.1 CD8^+^ T cell clones. Supernatants from the coculture were collected, and T cells were incubated with M113 target cells to assess their functions. All data are represented as mean (SD). (B) Cytotoxicity of T cells (clones WT4 and CTL03.1) measured by NLuc release from target M113-NLuc cells. *n* = 3 biological replicates. ∗*p* = 0.05 (Mann-Whitney test). (C) ELISA analysis of granzyme-B secretion induced by EV incubation, normalized to the spontaneous granzyme-B secretion by T cells that were not incubated with EVs. *n* = 4–8 biological replicates. ∗*p* = 0.0176 (Mann-Whitney test).

To investigate then the effect of EVs from OV-infected tumor cells on immune cells, we used two human CD8^+^ T cell clones that are specific for a peptide derived from the tumor antigen Melan-A in the HLA-A∗0201 context.^38^^,^^39^ The T cells were first incubated with EVs produced by melanoma cells before being cocultured with Melan-A^+^ target cells to evaluate their cytotoxic functions and phenotypic changes (Figure 5A). Viral titration of EV samples (Figure S7A) allowed us to estimate that T cells incubated with EVs-VSV were exposed to approximately 0.3 virion per cell in these series of experiments. However, it did not affect their viability (Figure S7B), nor did it lead to productive infection of T cells (Figure S7C).

We evaluated whether the cytotoxic properties of the two T cell clones WT4 and CTL03.1 were modified when they were exposed to EVs from either uninfected or OV-infected cells. As previously reported by our group,^12^ EVs from uninfected melanoma cells decreased the effector functions of T cells, with a varying impact depending on the effector:target ratios that were used (Figure 5B). However, when incubated with EVs-VSV, and to a lesser extent to EVs-VACV with the CTL03.1 clone, both T cell clones exhibited restored cytotoxic capacities similar to those observed in the untreated condition. Accordingly, we observed that the T cell clones exposed to EVs-VSV secreted higher amounts of granzyme-B compared with T cells exposed to EVs from uninfected melanoma cells (Figure 5C) and a slight—although not significant—increase in the surface expression of the degranulation marker CD107a (Figure S7D). However, we did not detect any change in TNF-α or IFN-γ secretion after incubation of T cells with EVs (Figure S7E). Overall, our results show that EVs-VSV partly increase the cytotoxic properties of human CD8^+^ T cells and may potentiate their anti-tumor properties.

## Discussion

In this study, we showed that OV infection of human tumor cell lines impacts EV biogenesis by increasing EV secretion and modifying their content. Our results suggest that the loading of viral proteins—either from the virus *per se* or from virus-encoded transgenes—may modify the properties of these EVs, in particular the efficacy of cargo transfer to uninfected cells. In addition, EVs-VSV are enriched in immunity-related proteins and were demonstrated to partly restore the cytotoxic functions of human Melan-A-restricted CD8^+^ T cell clones compared with EVs from uninfected tumor cells.

Our results broaden previous observations showing that products from oncolytic adenovirus transgenes can be packaged into vesicles^28^ and indicate that this phenomenon is shared with other viruses and in different cancer types. Given that proteins that we detected in EVs do not contain EV-addressing sequences, it is likely that the EV content simply reflects the cellular expression of the viral transgenes. EVs are easily accessible in most bodily fluids and are thus explored as biomarkers to follow disease progression or response to treatment.^40^^,^^41^^,^^42^ If detection of viral products in EVs indeed reflects the cellular content of infected cells, isolation of EVs from peripheral blood followed by biochemical quantification of proteins expressed by OVs could serve as a biomarker and be an alternative to the detection of viral genome to monitor OV replication within the tumor. It could also provide useful information on the cellular response to OV infection, for instance, regarding the innate immune response.

Intercellular protein transfer *via* EVs seems to be a rare event.^33^^,^^34^ We found that infection of tumor cells may alter this by (1) increasing EV secretion and (2) modifying the content of EVs, especially with the presence of fusogenic viral glycoproteins that would help endosomal escape and cytoplasmic cargo delivery in recipient cells. This has been reported by others,^34^^,^^35^^,^^43^ but we demonstrate here that fusogenic EVs can penetrate a 3D environment to deliver their cargo. In addition, our results on complex multicellular spheroids show that it is possible to deliver intracellular cargos to immune cells that can be found in tumors. Transgenic *in vivo* models, allowing the monitoring of tumor EV dissemination at basal state and upon treatment could provide very useful information to understand how OVs may alter intercellular communication in the tumor microenvironment.

Our preliminary mass spectrometry (MS) proteomic analysis detected 47 of the 50 proteins identified in the *Vesiclepedia* database as most often associated with EVs. According to the proposed markers for exosomes and ectosomes,^37^ OV infection does not seem to alter the ectosome/exosome ratio, even though one could expect VSV to increase ectosome secretion by assembly of viral particles at the plasma membrane. Conversely, even though VACV did not shift the ectosome/exosome ratio toward exosomes, MS results seem to indicate that the viral exploitation of vesicular transport^44^^,^^45^ has repercussions on EV content. Among proteins enriched in EVs-VACV, we did not detect proteins involved in the type I IFN response, which could be explained by the ability of VACV to evade innate immune signaling.^46^ Our results regarding the proteome of EVs-VSV matches previous work where the authors showed that the glioblastoma secretome after infection by oncolytic HSV-1 is linked to the immune reponse.^47^ However, the design of our study does not allow to determine whether the changes observed in the EV content reflect the cellular content or if the infection induces differential cargo loading during EV biogenesis. Nonetheless, our results suggest that EVs produced upon tumor infection by different OVs may present varying immunogenicity. Those may impact differently the surrounding environment, and it remains to be determined how EV-based intercellular communication in this context could positively or negatively impact surrounding normal cells.

Indeed, tumor-derived EVs have been previously described to interfere with immune cell functions. Even though tumor EVs can transfer antigens or functional peptide/MHC complexes to immune cells,^13^ their content is usually described as immunosuppressive.^5^^,^^12^^,^^48^ In a model of coculture of T cells with tumor EVs, our group previously observed that EVs from uninfected melanoma cells inhibited T cell cytotoxicity.^12^ Here, we report that EVs-VSV partly restore these cytotoxic functions, with an increase in both granzyme-B secretion and target cell lysis. It remains to be determined if this mechanism is related to proteins involved in the type I IFN response or other factors from innate immunity, or to antigen-specific mechanisms such as the EV-mediated presentation of functional MHC-peptide complexes to T cells. Upregulation of surface cellular MHC during OV infection is well known,^49^ and our results indicate that this is reflected in EVs, which could theoretically favor EV-mediated antigen presentation. Another imaginable explanation is that EVs-OV transfer whole tumor antigens to T cells, which are then delivered to their cytoplasm and subsequently processed to be presented to other T cells. This hypothesis is supported by the loading of the antigen Melan-A in EVs-VSV and the presence of fusogenic viral protein on EVs-OV, but this will need to be confirmed experimentally. Finally, T cell activation may be mediated by non-protein effectors, such as miRNAs, which we did not explore in this study.

The effects of tumor EVs from OV-infected cells on other cell types will need to be explored further to better understand how OVs modulate the tumor microenvironment. As an example, it was demonstrated that VSV-G is a TLR4 agonist and induces type I IFN secretion by mouse macrophages in a CD14-dependent, nuclear factor κB-independent manner.^50^ Moreover, VSV-G^+^ EVs induce DC maturation and cross-presentation *in vivo*.^51^ Therefore, it is likely that tumor EVs from infected cells have immunostimulatory properties for myeloid cells, which would further boost the anti-tumor immune response *in vivo*. However, in this immunogenic context the release of viral antigens may also enhance the adaptive antiviral immune response and could, thereby, limit the efficacy of viral-based therapies. EVs from infected cells could also influence directly the viral susceptibility of other tumor cells. As an example, EVs from cells infected by an oncolytic Newcastle disease virus (NDV) carry miRNAs that inhibit the type I IFN response and facilitate NDV replication and spread,^52^ but EVs can either promote or restrict infection depending on the context.^22^ How EVs influence the replication of other OVs will have to be studied as this could ultimately help to design viruses which exploit the right mechanisms for optimal viral replication and proper immune activation.

There is a growing interest in the field of therapeutic vectorization using EVs. Our results, together with others, also provides rationale for engineering OVs to exploit EVs and disseminate therapeutic proteins within the tumor microenvironment. Several articles have shown that biomolecules of interest (e.g., proteins, RNAs) can be targeted for loading into EVs, for instance by coupling them to tetraspanins,^53^ viral proteins,^35^^,^^54^ or with membrane-addressing, palmitoylation sequences, as used in our experiments. This allows the transfer of therapeutic molecules to the cytoplasm or the nucleus of the targeted—and bystander—cells. These systems could easily be adapted to OVs by inserting the retargeted sequences into their genomes,^21^ which could result in a two-step vectorization approach based on both OVs and EVs.

The presence of contaminant viral particles in EV preparations is a major concern when assessing their effects on immune cell functions. In this study, we were not able to completely eliminate VSV virions from EV preparations. Viral replication was not detected in T cells incubated with EVs-VSV, but we cannot completely rule out a role for these contaminants in our functional experiments. Regarding the existing literature, it is important to note that virions and EVs from virus-infected cells must have been co-purified and that we need to be cautious when attributing certain functions to one or the other.^47^^,^^55^ This also makes it very difficult to evaluate the specific role of EVs-OV *in vivo*. Moreover, several of our experiments suggest that EVs-VSV consist of a continuum of particles that share protein markers from both *bona fide* EVs and virions, which again complexifies our understanding of the mechanisms at play.

Overall, our results suggest that part of the activity of OVs could be mediated by a modification of the protein content of tumor EVs, which could have an impact on the anti-tumor immune response and antiviral immunity. Having access to blood samples of clinical cohorts of patients treated with OVs to observe the evolution of tumor EV content over time could help to better understand the mechanisms at play after actual OV treatments. Finally, our results advocate for the generation of recombinant OVs that exploit the EV machinery to better disseminate therapeutic proteins within the tumor microenvironment and modulate the activity of its different components.

## Materials and methods

### Plasmids and molecular biology

pMD2.G and psPAX2 were gifts from Didier Trono (Plasmids #12259 and #12260, Addgene, Watertown, MA). pUMVC was a gift from Bob Weinberg (Plasmid #8449, Addgene).^56^ pLV-CMV-LoxP-DsRed-LoxP-eGFP was a gift from Jacco van Rheenen (Plasmid #65726, Addgene).^8^ AAV-GFP/Cre was a gift from Fred Gage (Plasmid #49056, Addgene).^57^ pBS-N, pBS-P, pBS-L, and pVSV-XN2 were gifts from Richard Vile (Mayo Clinic, Rochester, MN).

To obtain pcDNA3.1-Palm-Cre-HiBiT, a plasmid encoding Cre fused to a palmitoylation sequence to increase its loading in EVs, Cre was amplified from AAV-GFP/Cre using fwCre_pcDNA-Palm and rvCre_pcDNA primers (Table 1) and cloned into the pcDNA3.1 backbone containing palmitoylation and HiBiT sequences (BamHI/NotI digestion followed by HiFi DNA assembly, New England Biolabs, Ipswich, MA). To obtain pMX2.1-NLuc, NLuc was amplified from pNL2.1[NLuc/Hygro] (Promega, Madison, WI) using fwNLuc_pMX and rvNLuc_pMX primers (Table 1) and cloned into the pMX2.1 backbone (EcoRI/HpaI digestion followed by HiFi DNA assembly).

**Table 1:** List of primers used for plasmid cloning

Primer name	Primer sequence
fwCre_pcDNA-Palm	5′-GCTCCGGAGGAGGAGGATCCtccaatttactgaccg-3′
rvCre_pcDNA	5′-CCAGGCGCTCGCGGCCGCatcgccatcttccagcag GCGC-3′
fwNLuc_pMX	5′-CAGTGTGGTGGTACGGGAATTCATGGTCTTC ACACTCGAAGATTTCG-3′
rvNLuc_pMX	5′-AATCTGGCTAGCTTAACAATTGCTAGTTAAC TTACGCCAGAATGCGTTCGC-3′

### Cell line generation and cell culture

Human melanoma cell lines (M6, M113, and M117) were obtained from tumor biopsies (Biocollection PC-U892-NL, CHU Nantes, France). Human malignant pleural mesothelioma (Meso4, Meso34, and Meso163) and human lung adenocarcinoma (ADCA153) cell lines were obtained from pleural effusions (Biocollection DC-2011-1399) and genetically characterized.^58^ Murine mesothelioma AK7 cell line was obtained as previously described.^59^ Other human lung adenocarcinoma (H441 and H1975), fibroblasts (HFF-2), monocytes (THP-1), mouse melanoma (B16/F1), and hamster BHK-21 cell lines were purchased from the American Type Culture Collection (LGC Standards, Middlesex, UK). The Lenti-X 293T cell line was purchased from Takara (Takara Bio Europe, Saint-Germain-en-Laye, France). M6-NLuc and M113-NLuc were obtained by retroviral transduction of pMX2.1-NLuc as previously described.^60^ [H441, H1975, HFF-2, M113, Meso34 and THP-1]-loxP-dsRed-loxP-GFP were obtained by lentiviral transduction of pLV-CMV-LoxP-DsRed-LoxP-eGFP and cultured with 1 μg/mL puromycin until complete selection of transduced cells. Human tumor cells were cultured in RPMI 1640 medium (Eurobio, Les Ulis, France). Murine tumor cells, Lenti-X 293T, HFF-2 and BHK-21 cells were cultured in DMEM (Gibco, Carlsbad, CA). Both media were supplemented with 10% heat-inactivated fetal calf serum (FCS, Corning, Corning, NY), 100 U/mL penicillin, 100 mg/mL streptomycin, and 2 mM L-glutamine (all from Gibco). For EV production experiments, medium was supplemented with EV-depleted FCS (100,000×*g*, 16 h).

CTL03.1 and WT4 T cell clones were obtained as previously described.^38^^,^^39^ CTL03.1 and WT4 are CD8^+^ T cell clones specific for HLA-A∗0201/Melan-A(26–35) (EAAGIGILTV). T cells were cultured in RPMI 1640 medium supplemented with 8% UltraGRO (AventaCell, Atlanta, GA), 100 U/mL penicillin, 100 mg/mL streptomycin, 2 mM L-glutamine, and 150 U/mL interleukin-2 (IL-2) (Proleukin, Novartis, Basel, Switzerland). For experiments, T cells were cultured in medium supplemented with 50 U/mL IL-2.

For tumor spheroid formation, 20,000 [H441 or H1975]-loxP-dsRed-loxP-GFP cells were seeded in 96-well U bottom Nunclon Sphera plates (Thermo Fisher Scientific, Waltham, MA). For complex spheroids, 20,000 Meso34 cells were mixed with 10,000 [THP-1 or HFF-2]-loxP-dsRed-loxP-GFP cells in 96-well U bottom Nunclon Sphera plates. The plates were centrifuged 2 min at 800×*g* and incubated for 3 days to allow spheroid formation.

All cells were cultured at 37°C in a 5% CO_2_ atmosphere and were routinely checked for mycoplasma contamination using PlasmoTest (Invivogen, San Diego, CA).

### OVs

Recombinant VSV encoding GFP (VSV-GFP) or NLuc (VSV-NLuc) between the *G* and *L* genes were generated from the wild-type Indiana strain by reverse genetics as previously described.^61^ Briefly, BHK-21 cells were infected with VACV MVA-T7 (a gift from Dr John Bell, Ottawa Hospital Research Institute, Ottawa, Canada) at a multiplicity of infection (MOI) = 1. Ninety minutes later, cells were transfected with pBS-N, pBS-P, pBS-L, and either pVSV-GFP or pVSV-NLuc. Two days later, supernatant was filtered at 0.22 μm to remove contaminating MVA-T7 and added to new BHK-21 cells to confirm VSV rescue. After amplification, VSV was purified by ultracentrifugation of the supernatant on a 10% sucrose cushion (100,000×*g*, 1 h). Viral titers were determined by plaque assay on BHK-21 cells.

Recombinant VACV was provided by Transgene SA (Illkirch-Graffenstaden, France). Briefly, it was derived from the Copenhagen strain deleted for *TK* and *RR* genes (VACV TK^−^RR^−^/GFP).^62^ VACV TK^−^RR^−^/GFP was propagated and titrated in chicken embryo fibroblasts, as previously described.^63^

### Analysis of viral infection and cell viability

For infection analysis, tumor cells were seeded at 7,000 cells per well in a 96-well plate and infected 3 days later with VSV-GFP or VACV-GFP (MOI 0.1 and 1). Plates were then cultured for 72 h in an Incucyte S3 (Sartorius, Goettingen, Germany) with images taken every 4 h for 3 days. Percentage of infection was determined with GFP expression by normalizing green integrated intensity with confluency (phase image).

Cell viability was determined using the CellTiter-Glo kit (Promega) according to the manufacturer’s instructions. Briefly, tumor cells were seeded at 7,000 cells per well in a 96-well plate and infected 3 days later with VSV-GFP or VACV-GFP (MOI 0.1 and 1). At 16 h, 24 h, 48 h, and 72 h post-infection, the CellTiter-Glo reagent was added into the wells (volume 1:1 with supernatants). After a 10-min incubation at 37°C, supernatants and lysed cells were transferred into a white-walled 96-well plate to measure luminescence with a Mithras LB 943 luminometer (Berthold Technologies GmbH, Bad Wildbad, Germany).

### EV production and isolation

For infection experiments, confluent tumor cells were infected at MOI = 0.1 (except M117, MOI = 0.05) and cultured in EV-depleted medium for 16 h. For transfection experiments, Lenti-X 293T cells were transfected with 1 μg DNA/million cells using Lipofectamine 3000 (Invitrogen, Carlsbad, CA). Six hours after transfection, medium was replaced with EV-depleted medium for 36 h. Conditioned media were centrifuged to remove cells (500×*g*, 5 min), cell debris (2,000×*g*, 10 min), large vesicles (10,000×*g*, 30 min), and filtered at 0.22 μm before ultracentrifugation to pellet EVs (100,000×*g*, 2 h). Pellets were washed with 0.22 μm-filtered PBS and ultracentrifuged a second time (100,000×*g*, 2 h). EVs were resuspended in 100 μL of 0.22 μm filtered PBS and either used immediately or after storage at −80°C. Ultracentrifugation was performed using an Optima L-80XP ultracentrifuge equipped with an SW 32 Ti rotor and open-top thinwall polypropylene tubes (all from Beckman Coulter, Indianapolis, IN).

### TEM and immunogold labeling

EVs were fixed in 2% paraformaldehyde (PFA) (Electron Microscopy Sciences, Hatfield, PA). Copper grids coated with a carbon film were effluxed overnight. Ten microliters of EVs were placed on the grid for 1.5 min before being quickly wiped on blotting paper. Negative staining was performed with 10 μL uranyl acetate (Agar Scientific, Stansted, UK) for a few seconds before being wiped on blotting paper. This operation was repeated twice. The grid was dried for a few minutes and then imaged with a JEOL JEM-1400 Plus (120 kV) transmission electron microscope (acquisition time = 1 s).

For immunogold labeling, formvar/carbon-coated nickel grids were deposited on a drop of samples during 5 min and rinsed twice with PBS. Grids were then incubated on a drop of PBS supplemented with 1% BSA and then PBS containing 1:100 anti-VSV-G and 1:100 anti-CD63 antibodies (antibody references in Table S2). After six 5-min washes with PBS, grids were further incubated for 1 h on a drop of PBS containing 1:30 gold-conjugated (6 nm) goat-anti-Rabbit IgG, and 1:30 gold-conjugated (10nm) goat-anti-Mouse (Aurion, Wageningen, the Netherlands). Grids were then washed with six drops of PBS, post-fixed in 1% glutaraldehyde and rinsed with three drops of distilled water. The negative staining was then performed with three consecutive contrasting steps using 2% uranyl acetate (Agar Scientific) before analysis under the transmission electron microscope (JEOL 1011, Tokyo, Japan).

### Flow cytometry analysis of EVs

EVs were stained for 30 min at 4°C with a mix of anti-tetraspanin (CD9, CD63, and CD81) antibodies or corresponding control isotypes conjugated to allophycocyanin (antibody references in Table S2). In some experiments, EVs were also stained with an anti-VSV-G antibody labeled with DyLight405 using a DyLight 405 Conjugation Kit (Abcam, Paris, France) according to the manufacturer’s instructions. To remove aggregates, antibodies were centrifuged at 15,000×*g* for 3 min before staining. Single EVs were then analyzed using an Attune NXT flow cytometer (Thermo Fisher Scientific).

### Western blot

Samples were lyzed with radio immunoprecipitation assay (RIPA) buffer (Sigma-Aldrich, Saint Louis, MO), incubated for 30 min at 4°C and centrifuged at 16,000×*g* for 30 min to pellet debris. Protein concentration was determined by bicinchoninic acid assay (BCA, Interchim, Los Angeles, CA) according to the manufacturer’s instructions. Unless otherwise indicated, equal amounts of proteins were mixed with Laemmli buffer (Bio-Rad, Hercules, CA) and heated at 95°C for 5 min. Proteins were separated on 4%–20% Bis-Tris gels (Genscript, Leiden, the Netherlands) at 200 V for 40 min and transferred onto PVDF membranes (Millipore, Burlington, MA) at 100 V for 60 min. For immunoblots, membranes were blocked in Tris-buffered saline 0.05% Tween 20, 5% fat-free milk for 1 h at room temperature and incubated overnight at 4°C with antibodies of interest (Table S3). Membranes were then incubated with horseradish peroxidase-conjugated secondary antibodies (Table S3) for 1 h at room temperature. Membranes were then incubated with Clarity western blotting substrate (Bio-Rad) and imaged with a ChemiDoc Imaging System (Bio-Rad). For detection of the HiBiT tag, the Nano-Glo HiBiT Blotting System (Promega) was used and membranes were processed according to the manufacturer’s instructions.

### Biochemical assays

#### Proteinase protection assay

EVs were diluted in PBS or Nano-Glo Luciferase Assay Buffer (Promega) and incubated with 22 μg/mL proteinase K (Macherey-Nagel, Düren, Germany) for 20 min at 37°C. The Nano-Glo Luciferase Assay Substrate (Promega) was added according to the manufacturer’s instructions and luminescence was acquired using a Mithras LB 943 luminometer (Berthold Technologies GmbH) with an acquisition time of 1 s.

#### EV internalization assay

NLuc^+^ EVs secreted by M113-NLuc were incubated with parental M113 for 4 h at 37°C. To remove unbound EVs, recipient cells were incubated in 0.05% trypsin-EDTA (Gibco) for 20 min at 37°C and washed twice with PBS. Nano-Glo Luciferase Assay Substrate (Promega) was added according to the manufacturer’s instructions and luminescence was acquired using a Mithras LB 943 luminometer (Berthold Technologies GmbH) with an acquisition time of 1 s. In parallel, luminescence of EVs used as input was measured in the same conditions and was used to normalize the luminescence of recipient cells.

#### Cre recombination assay

Transduced recipient cells were seeded as monolayers or spheroids and treated with a dose of Cre^+^ or Cre^+^/VSV-G^+^ EVs equivalent to a 10:1 secreting transfected cell:recipient tumor cell ratio. Cells were incubated for 3 days to allow for DNA recombination and GFP expression. For monolayer experiments, recipient cells were trypsinized, washed twice with PBS and analyzed using a FACSymphony A5 flow cytometer (BD Biosciences, Pont de Claix, France). For 3D experiments, spheroids were washed twice with PBS, fixed in PBS 4% PFA (Electron Microscopy Sciences) for 30 min at room temperature, washed twice with PBS and incubated for 24 h in PBS 0.5% Triton X-100 (Sigma-Aldrich) with 5 μg/mL Hoechst 33342 (Sigma-Aldrich). One day before observations, spheroids were cleared with Rapiclear 1.47 (Sunjin Lab, Hsinchu City, Taiwan) and mounted onto 15-well 3D μ-Slide (Ibidi GmbH, Gräfelfing, Germany) pre-coated with 25 μg/mL Cell-Tak cell and tissue adhesive (Corning). Spheroids were imaged using a Nikon A1rHD LFOV confocal microscope equipped with a 25×/1.05 oil immersion objective (Nikon Instruments, Tokyo, Japan). The pinhole size was set to 1 AU (15 μm), and the *Z*-interval between two slices was set to 2 μm. Images were processed and analyzed using Fiji.^64^ GFP^+^ and dsRed^+^ areas of each slice were segmented using the “Auto Threshold” plugin. The “Try all” option was first selected to empirically determine the optimal segmentation method. Images were subsequently analyzed with the following settings: “method = MaxEntropy white use_stack_histogram.”

### Protein MS

#### Liquid digestion of proteins

EVs were lysed with RIPA buffer (Sigma-Aldrich) and protein concentration was determined by BCA (Interchim) according to the manufacturer’s instructions. Proteins were first precipitated with ice-cold acetone for 1 h at −20°C. After centrifugation at 4°C for 15 min at 15,000×*g*, the protein pellets were prepared using the PreOmics iST kit (PreOmics GmbH, Planegg, Germany) following the manufacturer’s instructions. Briefly, samples were thawed and lysed (denatured, reduced, and alkylated) for 10 min at 95°C then Trypsin/LysC digested for 3 h at 37°C. Purification of peptides was then carried out at room temperature on spin cartridge and peptides were finally eluted in 10 μL of LC-load buffer. Peptide concentration were determined using the Pierce colorimetric quantitative peptide assays (Thermo Fisher Scientific) and 300 ng were injected into the TimsTOF Pro mass spectrometer (Bruker Daltonik GmbH, Bremen, Germany).

#### Data acquisition by nano liquid chromatography tandem mass spectrometry

Data acquisition by nano liquid chromatography tandem mass spectrometry (MS/MS) was performed as previously described.^65^ Briefly, the resulting peptide mixtures were separated on a 75 μm × 250 mm C18 IonOpticks Aurora 2 column (Ion Opticks Pty Ltd., Bundoora, Australia) with a NanoElute HPLC system (Bruker Daltonik GmbH) at a flow rate of 400 nL/min at 50°C. The separation was performed with a buffer gradient (buffer A: 0.1% formic acid, 98% H_2_O MilliQ, 2% acetonitrile; buffer B: 0.1% formic acid, 100% acetonitrile) for 120 min (2%–15% buffer B for 60 min; up to 25% at 90 min; up to 37% at 100 min; up to 95% at 110 min and finally 95% for 10 min to wash the column). The column was coupled in-line to a timsTOF Pro (Bruker Daltonik GmbH) with a CaptiveSpray ion source (Bruker Daltonik GmbH). LC-MS/MS data were acquired by the PASEF method^66^ with a total cycle time of 1.31 s, comprising 1 TIMS MS scan and 10 PASEF MS/MS scans. The 10 PASEF scans (100 ms each) contained, on average, 12 MS/MS scans per PASEF scan.

#### Protein identification

MS data were processed with the Data Analysis 5.1 software to produce the peak list of MS and MS/MS spectra (.mgf file). Peptide and protein identification was then performed using the Mascot database search engine (Mascot server v2.6.2; http://www.matrixscience.com) using its automatic decoy database search to calculate a false discovery rate (FDR) as previously described. MS/MS spectra were simultaneously compared with the UniProt KB UP000005640 (version of November 17, 2021) human proteome database restricted to one protein sequence per gene (20,588 sequences), to a viral proteome database (UP000002327 version of March 24, 2021 VSV proteome database + UP000008269 version of March 24, 2021 VACV proteome database; 263 sequences) and to a common proteomic contaminant database from the Max Planck Institute of Biochemistry (Martinsried, Germany) (247 sequences). The mass tolerance for MS and MS/MS was set at 15 pmm and 0.05 Da. The enzyme was set to full trypsin with allowed miscleavage. The modifications are fixed carbamidomethylation of cysteines, variable oxidation of methionine, variable acetylation of lysine and N-terminal proteins, and variable deamidation of asparagine and glutamine. Identification results from Mascot (.dat files) were imported into the Proline Studio software v2.1.2.^66^ This software was then used to validate the identification of proteins with a peptide rank = 1, an FDR of 1% on the peptide spectra match score and peptides with a minimum score of 30 (−10∗LOG10(*P*), where *P* is the absolute probability).

#### Relative quantification and pathway enrichment analysis

The Proline Studio software was also used to the spectral count comparison of the identified proteins in each samples as previously described.^67^ For each protein, a weighted spectral count is calculated, as suggested in Abacus,^68^ where shared peptides are combined and weighted according to the specific spectral counts of the different Protein Sets sharing the same peptide(s). To detect significant difference between samples, a beta-binomial test was performed on these weighted spectral counts and a *p* value was calculated for each Protein Set using the R package BetaBinomial 1.2 implemented in Proline Studio.^69^ Proteins that satisfied the following conditions were considered significantly enriched: adjusted *p* value of less than 0.05 and a fold-change of greater than 2. The Venn diagram was generated with the DeepVenn online tool.^70^ The enrichment analysis of Gene Ontology Biological Process pathways was performed in STRING (v11.0b)^71^ using the list of proteins significantly enriched in VSV or VACV samples as an input and with “Whole Genome” as statistical background. The fold enrichment of each pathway was calculated using this formula: Fold-enrichment = Number of observed proteins annotated to the pathway/Number of proteins expected to be observed in a random list of the same size.

### T cell functional experiments

T cell clones were incubated with a dose of EVs equivalent to a 10:1 secreting tumor cell:recipient T cell ratio for 24 h at 37°C. T cells were washed once with PBS, viable cells were numerated with a NucleoCounter NC-3000 (Chemometec, Allerod, Denmark) and used for subsequent functional experiments.

#### ELISA

Spontaneous granzyme-B secretion was measured in the supernatant of WT4 cells after 24 h of incubation with EVs using an ELISA granzyme-B kit (R&D Systems, Minneapolis, MN). Absorbance values at 450 nm and 570 nm were read using a Multiskan FC microplate reader (Thermo Fisher Scientific) and concentrations were calculated according to the manufacturer’s instructions.

#### Cytotoxicity assay

T cell clones (CTL03.1 or WT4) were cocultured with target cells (M113-NLuc) for 6 h at different effector:target ratios (20:1 and 40:1 for CTL03.1; 5:1 and 10:1 for WT4). Specific lysis was validated by coculture of T cell clones with a non-HLA-A∗0201 cell line (M6-NLuc). NLuc released in the supernatant was used as a readout for cytotoxicity. After incubation with the Nano-Glo Luciferase Assay Substrate (Promega) for 10 min at RT under agitation, supernatants were analyzed using a Mithras LB 943 luminometer (Berthold Technologies GmbH). Cytotoxicity was calculated using this formula: Cytotoxicity (%) = [NLuc release (RLU) − Spontaneous NLuc release (RLU) ∗ 100]/[Maximum NLuc release (RLU) − Spontaneous NLuc release (RLU)]. Spontaneous NLuc release was obtained by analyzing supernatant of target cells cultured alone. Maximum NLuc release was obtained by adding 100 μg/mL digitonin (Promega) to target cells cultured alone.

#### CD107a surface expression assay

WT4 cells were cocultured with target cells (M113) for 2 h at a 2:1 effector:target ratio in presence of 5 μM monensin (Sigma-Aldrich) and 8 μg/mL Alexa Fluor 647-conjugated anti-CD107a antibody (clone H4A3) (Biolegend, San Diego, CA). Cells were harvested, washed once with PBS, stained with 0.125 μg/mL R-phycoerythrin conjugated anti-CD8 antibody (clone HIT8a, BD Biosciences) for 30 min at 4°C, and analyzed using an Accuri C6+ flow cytometer (BD Biosciences).

### Statistical methods

Unless stated otherwise, data are represented as the mean ± SD of biological replicates. For EV diameter measurement, statistical significance was determined using a t test. For all other experiments, when comparing three or more groups, statistical significance was determined using the Kruskal-Wallis test followed by Dunn’s multiple comparison test. When comparing two groups, statistical significance was determined using the one-tailed Mann-Whitney test. ∗*p* value ≤ 0.05; ∗∗*p* value ≤ 0.01; ∗∗∗∗*p* value ≤ 0.0001. Statistical analyses were performed using Prism 8.0 (GraphPad Software, Boston, MA).

## Data and code availability

Liquid-based MS results are available online (Table S1). Other raw data and materials can be made available upon reasonable request to the corresponding author.

## Acknowledgments

This work was performed with financial support from ITMO Cancer of Aviesan on funds administered by Inserm, the 10.13039/501100001665French National Research Agency (ANR-20-CE18-0009), “La Ligue Régionale Grand-Ouest Contre le Cancer (CSIRGO),” and the ARSMESO44 foundation. The authors thank the cluster LUNG innOvatiOn (LUNG O2) for logistic support. This work was also supported by grants from Biogenouest, 10.13039/100015510Infrastructures en Biologie Santé et Agronomie (IBiSA), and Conseil Régional de Bretagne awarded to C.P. We acknowledge the following core facilities for their technical expertise and help: IBISA MicroPICell facility (Biogenouest), member of the national infrastructure France-Bioimaging supported by the 10.13039/501100001665French National Research Agency (ANR-10-INBS-04); Cytocell - Flow Cytometry and FACS core facility (SFR Bonamy, BioCore, Inserm UMS 016, CNRS UAR 3556, Nantes, France), member of the Scientific Interest Group (GIS) Biogenouest and the Labex IGO program supported by the 10.13039/501100001665French National Research Agency (n°ANR-11-LABX-0016-01); Imp@ct core facility (SFR Bonamy, Nantes Université, CNRS, Inserm, Nantes, France), member of the Scientific Interest Group Biogenouest; IBISA electron microscopy core facility (PST Analyze des systèmes biologiques, Université de Tours, Tours, France). We thank John Bell for providing MVA-T7. We thank Richard Vile for providing plasmids used for VSV rescue. We thank Virginie Dehame, Jéromine Samain, and Emma Soussens for their technical help. Schematics were created with BioRender.

## Author contributions

Conceptualization: U.H., T.P., and N.B.; Funding acquisition: N.B., D.F., T.P., and J.-F.F.; Investigation: U.H., C.G., M.K., M.F, J.F., B.A., E.C., G.C, J.B-G, T.P., and N.B.; Supervision: J.-F.F., N.B., D.F., P.E., N.L., and C.P.; Writing – original draft: U.H.; Writing – review & editing: J.-F.F. and N.B.

All authors have read and approved the manuscript.

## Declaration of interests

P.E. is an employee of Transgene SA. Transgene SA is a member of the Institut Mérieux Group, a publicly traded French biopharmaceutical company.

## Supplemental information

Document S1. Figures S1–S7, Tables S2 and S3, and Supplemental materials and methods

Table S1. Relative quantification data of proteins identified in EVs by liquid-based mass spectrometry

Document S2. Article plus supplemental information
