# Supplementary material for: Oncolytic viruses alter the biogenesis of tumor extracellular vesicles and influence their immunogenicity
Source: Mol Ther Oncol. 2024 Sep 26;32(4):200887. doi: 10.1016/j.omton.2024.200887 (PMC11530755; doi:10.1016/j.omton.2024.200887)
Supplement: Document S1. Figures S1–S7, Tables S2 and S3, and Supplemental materials and methods [file mmc1.pdf]

OMTON, Volume *32*

Supplemental information

Oncolytic viruses alter the biogenesis of tumor extracellular vesicles and influence their immunogenicity

Ugo Hirigoyen, Coraly Guilbaud, Morgane Krejbich, Morgane Fouet, Judith Fresquet, Bastien Arnaud, Emmanuelle Com, Charles Pineau, Gwenann Cadiou, Julien Burlaud-Gaillard, Philippe Erbs, Delphine Fradin, Nathalie Labarrière, Jean-François Fonteneau, Tacien Petithomme, and Nicolas Boisgerault

Supplemental figures and tables

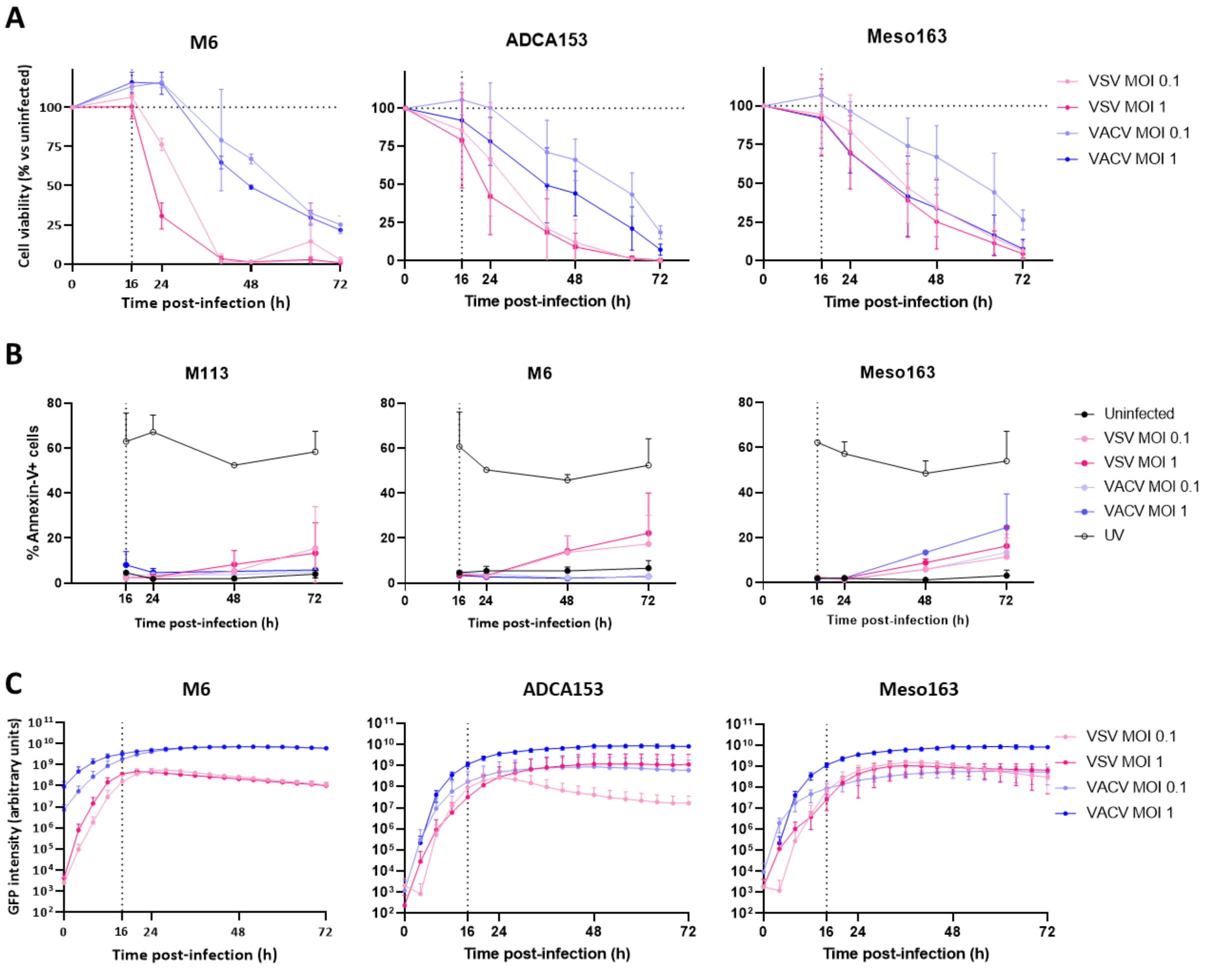

Figure S1. Analysis of cell death and GFP expression after OV infection.

(A-C) M6 and M113 melanoma cells, ADCA153 lung adenocarcinoma cells and Meso163 pleural mesothelioma cells were infected by VSV-GFP or VACV-GFP at MOI = 0.1 or 1. Cell viability (A), apoptosis (B) and expression of the recombinant GFP (C) were measured over time. Dotted lines indicate when EVs were harvested. Data are presented as mean (SD). n = 3 to 4 biological replicates.

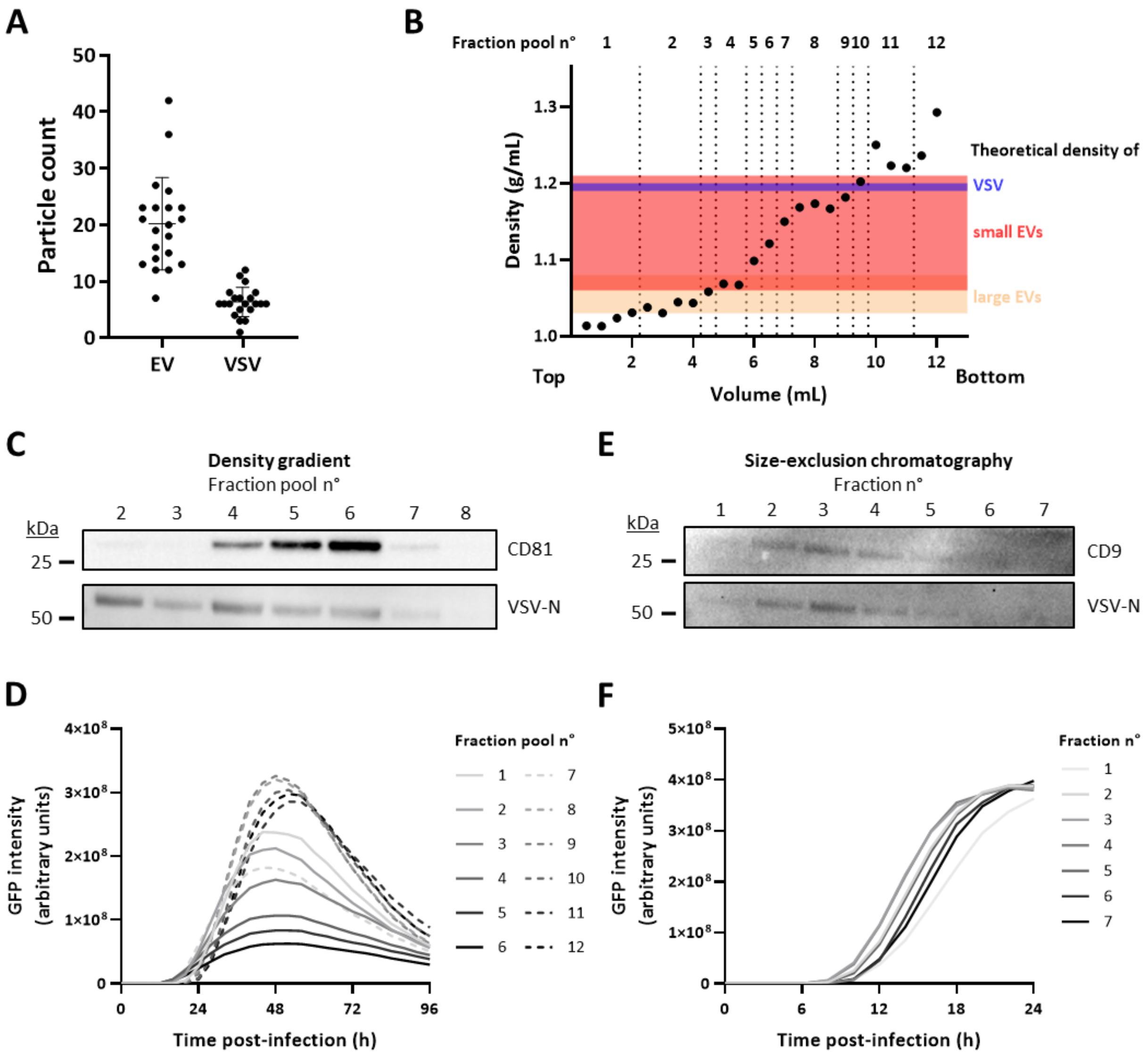

Figure S2. EVs from VSV-infected tumor cells copurify with virions.

(A) Quantification of spherical (EVs) or bullet-shaped (virions) particles observed in transmission electron micrographs. Data are represented as mean ± SD. n = 21 images. (B-D) Density floatation assay of EVs from M113 cells infected by VSV. (B) Density of individual fractions collected measured by 340 nm absorbance. (C) Western-blot analysis of CD81 and VSV-N after pooling of fractions with similar densities. (D) Analysis of GFP expression by BHK-21 cells incubated with pooled fractions to follow viral infection. (E-F) Size-exclusion chromatography of EVs from M113 cells infected by VSV. (E) Western-blot analysis of CD9

and VSV-N in eluted fractions. (F) Analysis of GFP expression by BHK-21 cells incubated with eluted fractions to follow viral infection.

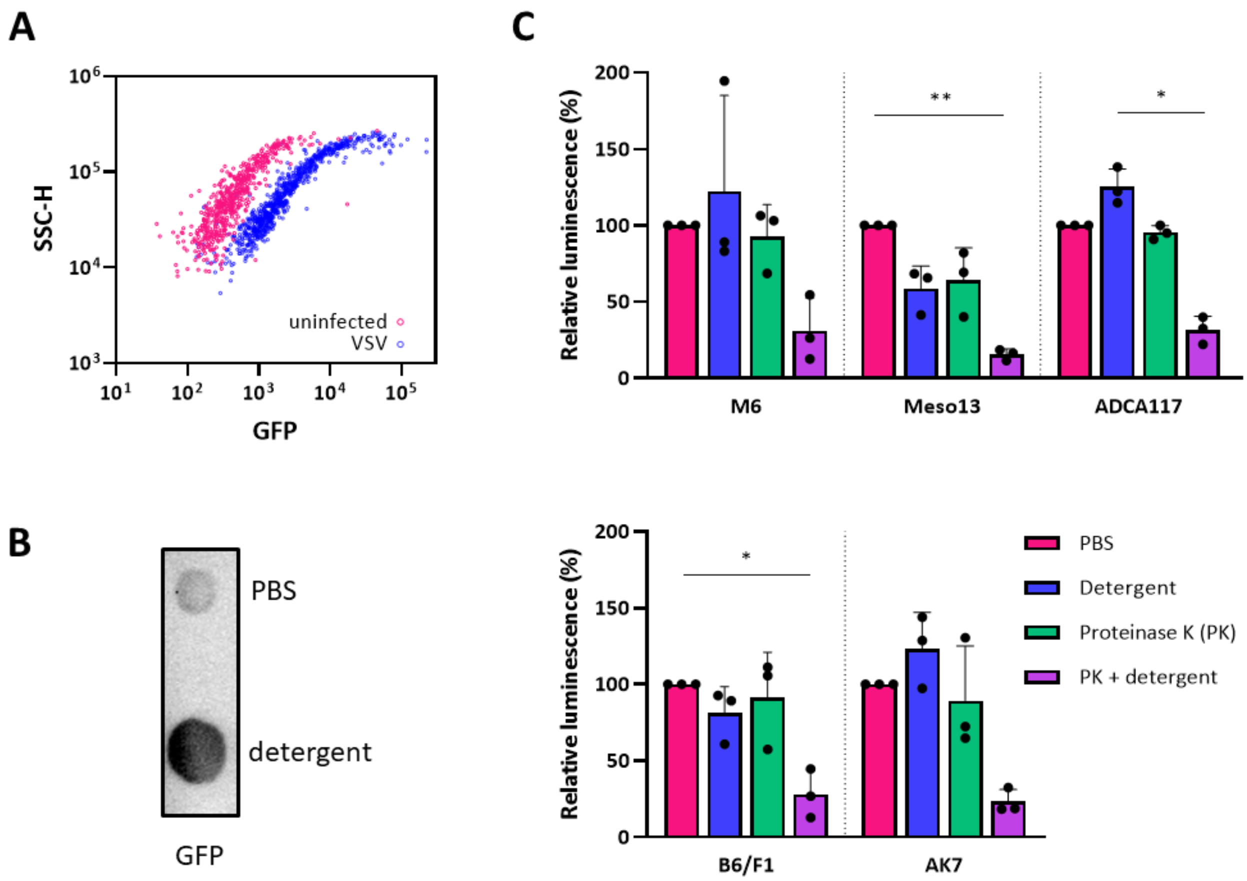

Figure S3. Analysis of the presence of recombinant proteins in EVs-VSV.

(A) Single-EV flow cytometry analysis of GFP in EVs-VSV secreted by M113 cells. EVs were gated based on the forward and side scatter parameters. (B) Dot blot analysis for detecting GFP in EVs lysed or not with detergent. (C) Proteinase protection assay of EVs secreted by M6 (melanoma), Meso13 (pleural mesothelioma), ADCA1117 (lung adenocarcinoma), B16/F1 (murine melanoma) or AK7 (murine mesothelioma) cells infected by VSV-NLuc. EVs were incubated with detergent, proteinase K or both before adding the NLuc substrate. Data are represented as mean (SD). n = 3 biological replicates. * p-value < 0.05 and ** p-value < 0.01 (Kruskal-Wallis test).

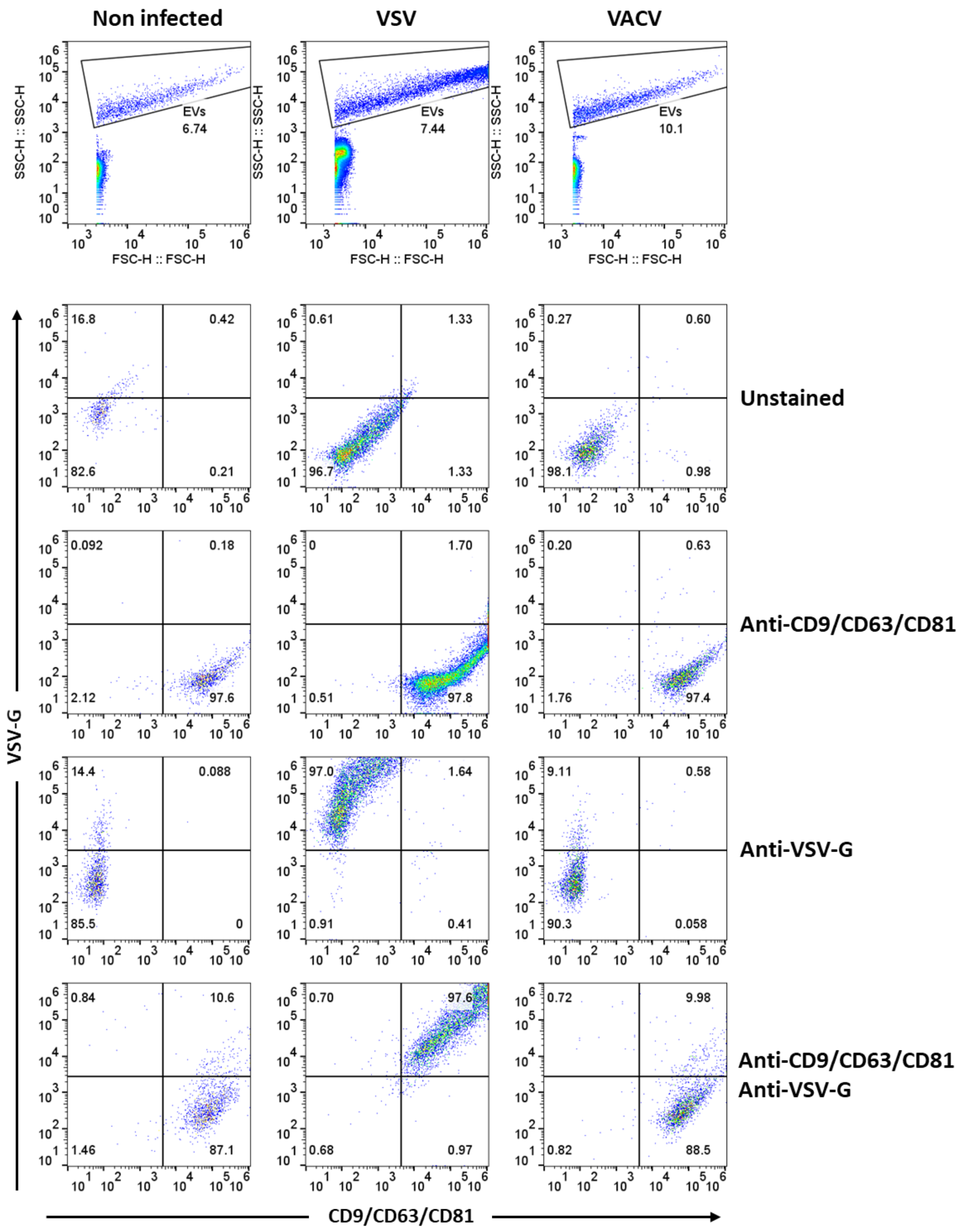

Figure S4. Analysis of EV surface markers by single-EV flow cytometry.

EVs secreted by uninfected, VSV-infected or VACV-infected M113 cells were stained for CD9/CD63/CD81 and VSV-G and analyzed by single-EV flow cytometry. They were first

selected based on the side scatter and forward scatter parameters. Single stainings were performed across conditions to verify specificity. Representative of 3 biological replicates.

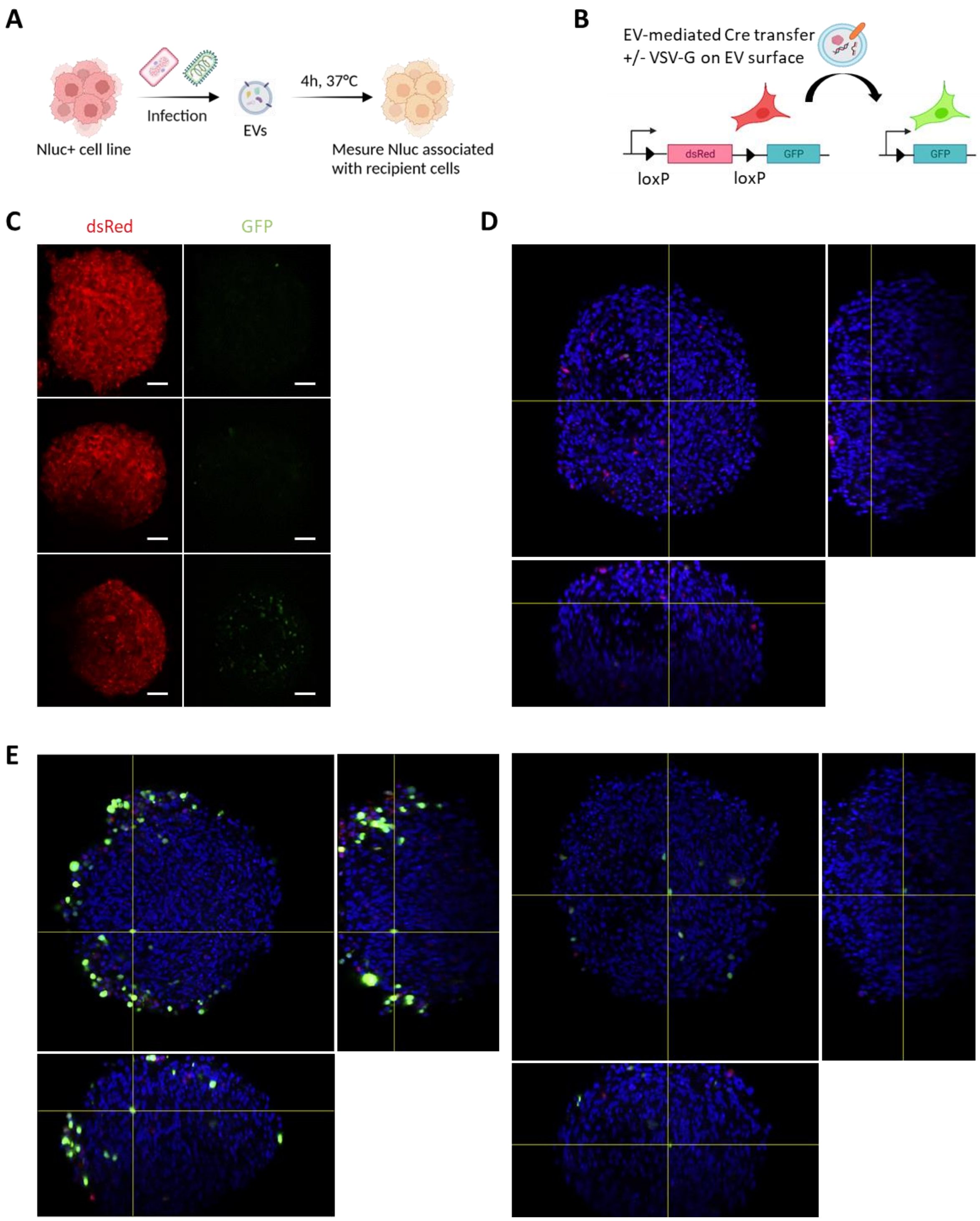

Figure S5. Analysis of the transfer of EV cargos.

(A) Experimental setup to study the intercellular transfer of NLuc. EVs secreted by M113-NLuc cells were incubated with parental M113 cells. EV internalization by recipient cells was measured by analyzing luminescence of target cells, normalized with input EV luminescence.

(B) Experimental setup to study the intercellular transfer of Cre. EVs of Lenti-X 293T transfected to express Cre and/or VSV-G were incubated with loxP-dsRed-loxP-GFP tumor cells transduced to express GFP upon Cre recombination. (C) Confocal micrographs of tumor spheroids (dsRed/GFP H441 cells) incubated with mock, Cre^{+} or Cre^{+}/VSV-G^{+} EVs. Images represent the median slice of the spheroid. Scale bar represents 100 µm. (D-E) Orthogonal views of Meso34(unlabeled)/THP-1(dsRed/GFP) spheroids treated with Cre^{+} EVs (D) or Cre^{+}/VSV-G^{+} EVs (E). Blue = DAPI.

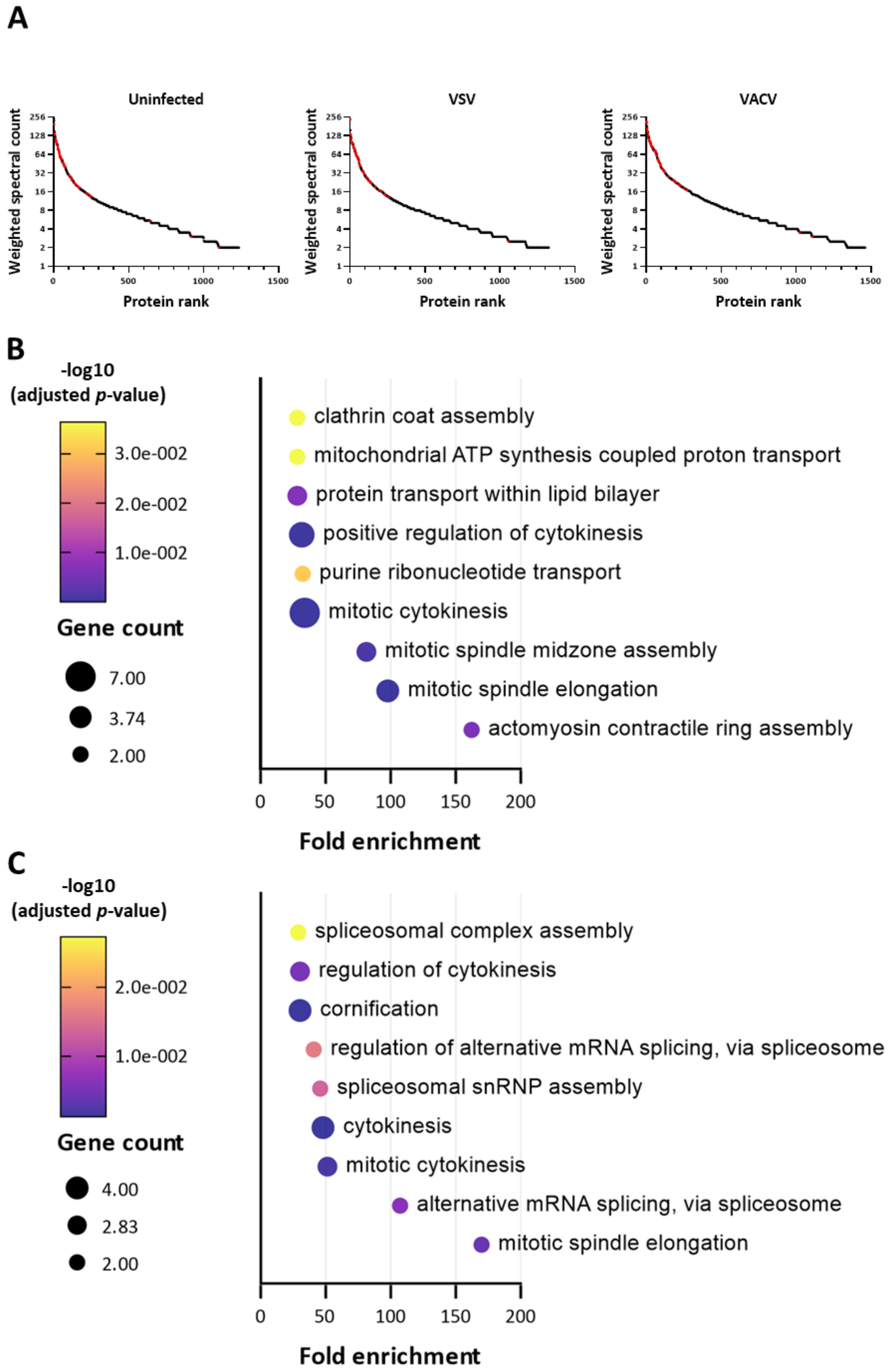

Figure S6. Additional results obtained by mass spectrometry.

(A) Relative quantification score (weighted spectral count) of individual proteins identified in samples. Proteins most commonly identified in Vesiclepedia are highlighted in red. (B-C) Significantly downregulated Gene Ontology (GO) biological pathways in EVs-VSV (B) and EVs-VACV (C).

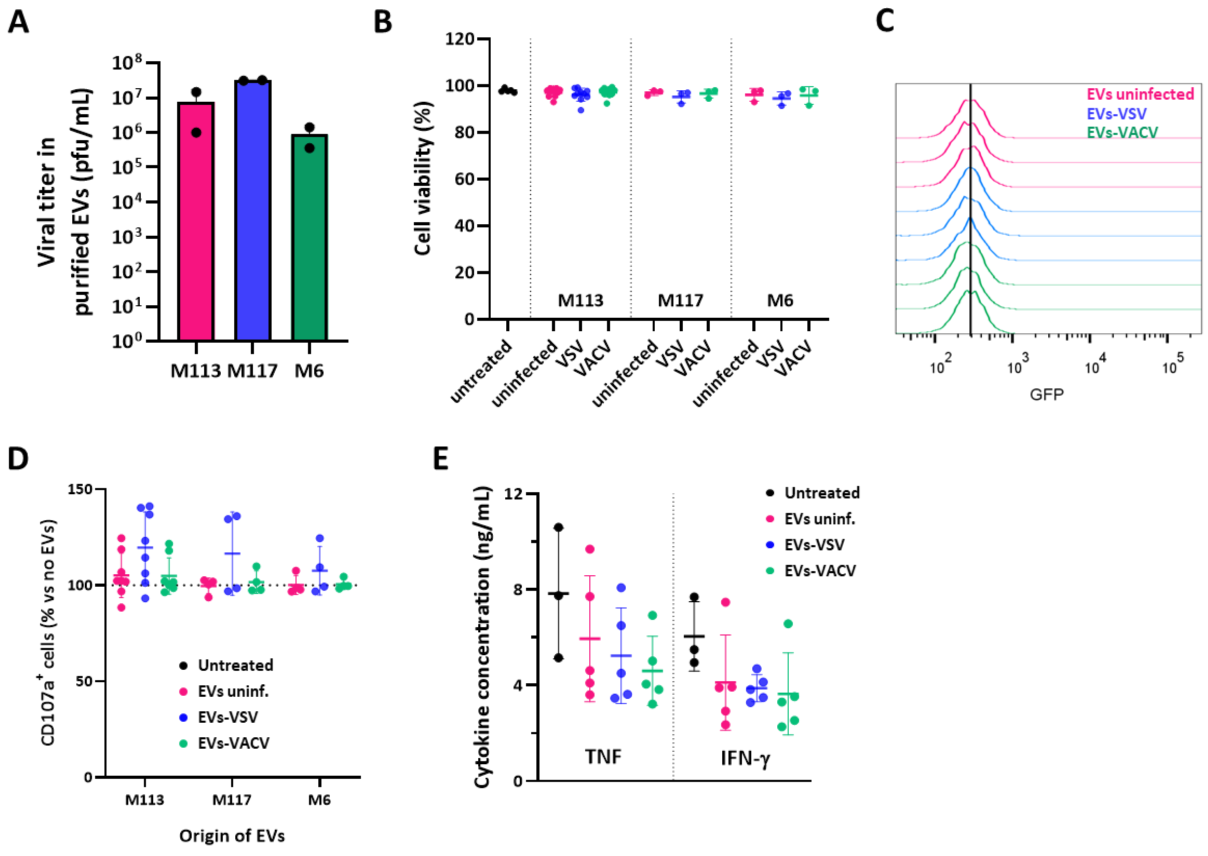

Figure S7. EVs from infected tumor cells do not fully rescue T cell functions.

(A) Viral titration by plaque-forming assay of EVs samples from cells infected by VSV. Data are represented as mean (SD). n = 2 biological replicates. (B) Cell viability of WT4 clone after 24 h of incubation with EVs. Data are represented as mean (SD). n = 3 to 10 biological replicates. (C) Flow-cytometry analysis of GFP in WT4 T cells incubated for 24 h with EVs from uninfected, VSV-infected or VACV-infected M113 cells. n = 3 biological replicates. (D) EVs secreted by M6 (HLA-A2^{neg}), M113 (HLA-A2^{+}) or M117 (HLA-A2^{+}) cells were incubated with HLA-A2-restricted CTL03.1 or WT4 CD8+ T cell clones. (E) ELISA quantification of TNF and IFN-γ secretion by T cells pre-incubated with EVs for 24 h and cocultured with target cells for 6 h. Data are represented as mean (SD). n = 3 to 5 biological replicates.

Table S1. Relative quantification data of proteins identified in EVs by liquid-based mass-spectrometry.

Please see attached .xlsx file.

Table S2. List of antibodies used for EV flow cytometry analysis.

Antibody specificity (clone number)	Working concentration	Catalog number
CD9 (REA1071)	0.125 µg/mL	130-118-867 (Miltenyi Biotec)
CD63 (REA1055)	0.19 µg/mL	130-127-390 (Miltenyi Biotec)
CD81 (REA513)	0.75 µg/mL	130-119-825 (Miltenyi Biotec)
Control isotype (REA293)	Same as relevant antibody	130-113-438 (Miltenyi Biotec)
VSV-G (8G5F11)	1 µg/mL	Ab01401-23.0 (Absolute Abs)

Table S3. List of antibodies used for western-blot.

Antibody specificity (clone number)	Working concentration	Catalog number
ALIX (3A9)	1 µg/mL	634502 (Biolegend)
β-actin (C4)	0.2 µg/mL	MA5-11869 (Invitrogen)
β2-microglobulin (B2M-01)	0.5 µg/mL	1X-237-C025 (EXBIO)
BST2 (E-4)	0.4 µg/mL	sc-390719 (Santa-Cruz)
Calnexin (AF18)	1 µg/mL	MA3-027 (Invitrogen)
CD9 (1021007)	1 µg/mL	MAB25292 (R&D systems)
CD63 (Ts63)	0.5 µg/mL	10628D (Invitrogen)
CD81 (M38)	0.5 µg/mL	10630D (Invitrogen)
GFP (13.1)	0.4 µg/mL	1181446001 (Roche)
HLA-ABC (W6/32)	Unknown concentration, 1:500 dilution	M0736 (DAKO)
Melan-A (A103)	0.2 µg/mL	sc-20032 (Santa Cruz)
VSV-G (8G5F11)	1 µg/mL	EB0010 (Kerafast)
VSV-N (10G4)	1 µg/mL	EB0009 (Kerafast)
Goat anti-mouse HRP-conjugated secondary antibody	Unknown concentration, 1:2000 dilution	115-036-072 (Interchim)
Goat anti-rabbit HRP-conjugated secondary antibody	Unknown concentration, 1:5000 dilution	111-035-006 (Interchim)

Supplemental materials and methods

Analysis of cell death

Cell death was determined by staining cells with Annexin-V. Briefly, tumor cells were seeded at 40,000 cells per well in a 24-well plate and infected 3 days later with VSV-GFP or VACV-GFP (MOI 0.1 and 1). Cells were exposed to UV-B (312 nm, 10 J/cm², Bio-Link, Vilber Lourmat) as a positive control. At 16h, 24h, 48h and 72h post-infection, the cells were recovered and stained with 1µL of Annexin-V-APC (BD Biosciences, Pont De Claix, France) in binding buffer as recommended by the manufacturer. After a 15-minute incubation, cells were resuspended in fresh PBS and analyzed by flow cytometry (BD Accuri). Data were analyzed using the FlowJo 10 software (BD Biosciences, Pont De Claix, France).

Density flotation assay

EVs were first concentrated using the same ultracentrifugation protocol as used throughout this study. Pellets were resuspended in PBS 4 % iodixanol (Sigma-Aldrich, Saint-Louis, MO), overlayed onto a iodixanol gradient (Figure S1B) and ultracentrifuged at 100,000 × *g* for 16 h. Fractions of 500 µL were collected and their density was determined by measuring absorbance at 340 nm of a control gradient, as described elsewhere.[1] Fractions of equivalent densities were pooled as indicated in Figure S1B. Pooled fractions were then diluted 10-fold in PBS and ultracentrifuged at 100,000 × *g* for 4 h. Ultracentrifugation was performed using an Optima LE-80 ultracentrifuge equipped with a SW 41 Ti rotor and open-top thinwall Ultra-Clear tubes (all from Beckman Coulter, Indianapolis, IN). EVs were resuspended in 100 µL of 0.22 µm filtered PBS and used for western-blot analysis or infectivity assay.

Size-exclusion chromatography

EVs were first concentrated using the same ultracentrifugation protocol as used throughout this study. Size-exclusion chromatography was then performed according to the manufacturer's instructions. Briefly, EVs were loaded onto qEV Original 70 nm columns (IZON, Christchurch, New Zealand) and elution fractions of 250 µL were collected after a 3 mL default buffer volume. Elution fractions were ultracentrifuged at 100,000 × *g* for 2 h, EVs were resuspended in 100 µL of 0.22 µm filtered PBS and used for western-blot analysis or infectivity assay.

Infectivity assay

Co-isolation of virions with EVs after flotation assay or size-exclusion chromatography was assessed by incubating BHK-21 cells with the different fractions. Infection of BHK-21 was assessed by monitoring GFP expression using an Incucyte S3 system (Sartorius GmbH, Goettingen, Germany) with the following settings: 4 × objective, 300 ms acquisition time in green channel, scan every 2 h (size-exclusion chromatography experiment) or 4 h (flotation assay experiment). GFP expression was calculated using the total integrated intensity measured in the green channel (green calibrated unit × μm^2).

Plaque-forming assay

VSV from EV preparations was titered by incubating BHK-21 cells with serial dilutions of samples. After 1 h of incubation to allow infection of cells, medium was removed, cells were washed once with PBS and overlayed with medium containing 1 % noble agar (Sigma-Aldrich, Saint-Louis, MO). Plates were kept at room temperature for 15 min to allow agar solidification and incubated for 1 to 2 days at 37 °C. Viral titers were determined by counting GFP^+ plaques in wells corresponding to at least 2 serial dilutions.

T cell functional experiments

T cell viability

After incubation with EVs for 24 h, WT4 T cells were stained using AO/DAPI (Chemometec, Allerod, Denmark) according to the manufacturer's instructions and viable cells were quantified with a NucleoCounter NC-3000 (Chemometec, Allerod, Denmark).

ELISA assay

TNF-α and IFN-γ secretion by WT4 cells cultured with target cells for 6 h was quantified using an ELISA TNF or IFN-γ kit (Invitrogen, Carlsbad, CA). Absorbance values at 450 nm and 570 nm were read using a Multiskan FC microplate reader (Thermo Scientific, Waltham, MA, USA) and concentrations were calculated according to the manufacturer's instructions.

Supplemental references

1. Kugeratski, F.G., Hodge, K., Lilla, S., McAndrews, K.M., Zhou, X., Hwang, R.F., Zanivan, S., and Kalluri, R. (2021). Quantitative proteomics identifies the core proteome of exosomes with syntenin-1 as the highest abundant protein and a putative universal biomarker. Nat. Cell Biol. *23*, 631–641. 10.1038/s41556-021-00693-y.
